# A Simple and Fast Protocol to Detect Nucleophosmin 1 (*NPM1*) Mutation and Fms-like Tyrosine Kinase 3 Internal Tandem Duplication (*FLT3*/ITD): Optimizing Laboratory Routine

**DOI:** 10.3390/mps9020059

**Published:** 2026-04-03

**Authors:** Alessandra Helena da Silva Hellwig, Gisele Menezes Ewald, Grazielle Motta Rodrigues, William Latosinski Matos, Juliana Bergmann, Viviane Horn de Melo, Rodrigo Minuto Paiva, Dariane Castro Pereira, Eduardo Wandame Gomez

**Affiliations:** 1 Residência Multidisciplinar em Área Profissional Análises Clínicas, Hospital de Clínicas de Porto Alegre, Porto Alegre 90035-903, Brazil; ahellwig@hcpa.edu.br (A.H.d.S.H.); gmorodrigues@hcpa.edu.br (G.M.R.); wlmatos@hcpa.edu.br (W.L.M.); egomez@hcpa.edu.br (E.W.G.); 2 Programa de Pós-Graduação em Medicina Ciências Médicas, Universidade Federal do Rio Grande do Sul, Porto Alegre 90035-903, Brazil; 3 Unidade de Imunologia de Transplantes e Medicina Personalizada, Serviço de Diagnóstico Laboratorial, Hospital de Clínicas de Porto Alegre, Porto Alegre 90035-903, Brazil; gewald@hcpa.edu.br; 4 Unidade de Microbiologia, Setor de Biologia Molecular, Serviço de Diagnóstico Laboratorial, Hospital de Clínicas de Porto Alegre, Porto Alegre 90035-903, Brazil; jbergmann@hcpa.edu.br (J.B.); vdmelo@hcpa.edu.br (V.H.d.M.); rpaiva@hcpa.edu.br (R.M.P.)

**Keywords:** multiplex polymerase chain reaction, capillary electrophoresis, nucleophosmin 1, fms-like tyrosine kinase 3, acute myeloid leukemia

## Abstract

Acute myeloid leukemia (AML) is a heterogeneous hematologic malignancy. AML classification is currently based on cytogenetic and molecular alterations as well as immunophenotyping, although risk stratification still relies primarily on cytogenetic findings. However, approximately 45% of AML patients present with a normal karyotype, which makes accurate risk classification and treatment stratification more challenging. Therefore, the identification of molecular prognostic markers described in the literature has become essential in routine diagnostic laboratories, enabling the more precise categorization of patients into risk groups. In this study, we present a simple, rapid, step-by-step multiplex PCR protocol combined with capillary electrophoresis for the detection of two of the most prevalent molecular alterations in AML: nucleophosmin 1 (*NPM1*) mutations and Fms-like tyrosine kinase 3 internal tandem duplications (*FLT3*/ITD). This protocol provides a practical workflow that can assist diagnostic laboratories in implementing and optimizing multiplex mutation detection in routine practice.

## 1. Introduction

Acute myeloid leukemia (AML) is a clinically heterogeneous hematologic malignancy [1,2,3,4], both in biological and morphological aspects [2,3], despite the relatively low number of genomic alterations when compared to other cancers [4]. The karyotype of patients with AML is widely studied; however, this isolated analysis does not provide sufficient information for prognostic stratification, since approximately 45% of AML patients present with a normal karyotype, requiring additional molecular analyses [2,3].

AML is characterized by a complex molecular landscape involving recurrent mutations in genes such as *NPM1*, *FLT3*, *TP53*, *ASXL1*, *DNMT3A* and *IDH1/2*, which play important roles in prognosis, risk stratification and therapeutic decision making [5]. The gene responsible for the synthesis of nucleophosmin (NPM) is highly conserved among humans [4]. It is located on chromosome 5q35 and is composed of 12 exons [2,3,4,5,6]. NPM1 is a protein that resides in the nucleolus under normal physiological conditions, but continuously shuttles between the nucleus and the cytoplasm [2,3,4,6]. NPM1 is a multifunctional protein [2] directly involved in the regulation and stability of nuclear proteins [3], playing an important role in the cell cycle and participating in deoxyribonucleic acid (DNA) repair processes [2,6].

Mutations in the *NPM1* gene are found in around 45–55% of AML patients with a normal karyotype and typically consist of the insertion of four nucleotides in exon 12 [1,3,7], causing the abnormal localization of the NPM1 protein in the cytoplasm [3,8]. These mutations are classified from types A to F; however, type A mutations are the most prevalent, occurring in approximately 75–80% of cases, followed by other variants such as types B and D [2,6,8].

Another important gene involved in AML pathogenesis is Fms-like tyrosine kinase-3 (FLT3), located on chromosome 13q12 [2,3]. This gene encodes a receptor with tyrosine kinase activity associated with cell signaling pathways [3,9], which, when activated, leads to increased proliferation and the inhibition of apoptosis [2]. Mutations in this gene result in structural changes in the FLT3 receptor, producing a constitutively active receptor that promotes the uncontrolled proliferation of myeloid cells [2]. FLT3 mutations may occur as point mutations or internal tandem duplications (ITD), the latter being among the most frequent mutations in AML (approximately 35–45%) and typically involving exons 14 or 15 [2,3]. *FLT3*/ITD mutations consist of duplications of DNA sequences that can range from 3 to more than 400 base pairs, most commonly occurring as in-frame insertions (multiples of three nucleotides) [10,11].

From a prognostic perspective, genetic mutations serve as important molecular markers in the diagnostic routine for AML patients. For example, *NPM1* mutation in the presence of normal cytogenetics is associated with favorable prognosis, whereas *FLT3*/ITD is associated with adverse prognosis (intermediate risk irrespective of the allelic ratio or presence of NPM1 mutation) [12].

Accurate AML diagnosis requires the integration of clinical, biological, cytogenetic, immunophenotypic and molecular data [1,2]. Therefore, the detection of mutations in the *FLT3* and *NPM1* genes has become an essential component of routine molecular diagnostics, providing important information related to the relapse risk, treatment response and disease-free survival [1,2,9,12,13].

Although fragment analysis for the detection of *NPM1* exon 12 insertions and *FLT3*/ITD mutations is already well established in molecular hematology laboratories, the implementation of multiplex workflows often relies on locally adapted procedures. The aim of this work is not to introduce a new detection method, but to provide a simple, reproducible and implementation-oriented protocol that facilitates workflow standardization, training and routine molecular diagnostic testing in AML. Here, we present a simple, rapid and cost-effective multiplex protocol for the detection of the most prevalent *NPM1* mutations (types A, B and D) and *FLT3*/ITD, enabling the implementation and optimization of laboratory routines using multiplex PCR and capillary electrophoresis.

## 2. Materials and Methods

### 2.1. Experimental Design

**Figure 1:**
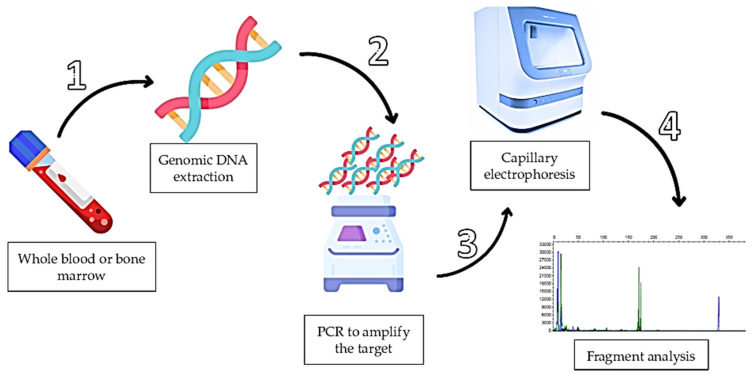
Summary of the mutation detection workflow using a simple and easy protocol. Genomic DNA extraction was performed from whole blood or bone marrow and amplified by conventional PCR. The PCR products were analyzed using a genetic analyzer and fragment analysis was performed based on the resulting electropherograms. The image was created with Flaticon.com (accessed on 12 November 2023).

The assay described here for detection of *NPM1* mutations (types A, B and D) and *FLT3*/ITD in AML samples is currently implemented in our laboratory routine. *FLT3* point mutations, including tyrosine kinase domain (TKD) mutations, are not detected by this method. A schematic overview of the workflow is presented in Figure 1. The protocol consists of four main steps: genomic DNA extraction from whole blood or bone marrow samples, amplification of target regions by conventional PCR, capillary electrophoresis using a genetic analyzer and fragment analysis for mutation detection.

One important limiting factor of this technique is the proportion of leukemic blasts present in the biological sample, since higher blast percentages generally increase the sensitivity of mutation detection. Although a specific blast-percentage cutoff is no longer required for AML with defined genetic abnormalities [12], during protocol implementation, mutation detection was successfully achieved in a specimen containing approximately 8% blasts.

As this protocol relies on PCR amplification followed by fragment analysis, the method is optimized for insertions that produce measurable size differences relative to the wild-type amplicon. Rare or atypical variants—such as uncommon insertion sizes, complex insertions or deletions or variants outside the amplified region—may not be detected by fragment analysis alone and complementary molecular methods may be required when atypical electropherogram patterns are observed. This approach is intended primarily for mutation detection and risk stratification, whereas minimal residual disease (MRD) monitoring requires higher-sensitivity methodologies, such as quantitative PCR or sequencing-based approaches [5].

A summary of the analytical validation performed in our laboratory, including sample distribution, specificity, concordance and precision, is provided as Supplementary Materials. However, laboratories implementing this in-house assay are expected to perform their own validation according to local regulatory and accreditation requirements.

### 2.2. Materials

Please note, all supplies and reagents used should be DNA- and RNA-free.

#### 2.2.1. Genomic DNA Extraction

1. Maxwell^®^ RSC Blood DNA Kit (Promega Corporation, Madison, WI, USA; Cat. no.: AS1400);
2. Pipettes and sterile tips;
3. Eppendorf tubes 1.5 and 2 mL.

#### 2.2.2. Polymerase Chain Reaction

1. Primer mix (forward and reverse) for detection of *NPM1* mutations (Table 1);
2. Primer mix (forward and reverse) for detection of *FLT3*/ITD mutations (Table 1);

3. Taq DNA Polymerase 5 U/μL 500 U, buffer 10× and MgCl_2_ 50 mM (Ludwig Biotecnologia, Alvorada, RS, Brazil; Cat. no.: 35);
4. Eppendorf tubes 0.2 mL;
5. Nuclease-free water for molecular biology;
6. dNTP set 100 mM (QuatroG Biotecnologia, Porto Alegre, RS, Brazil; Cat. no.: 100016).

#### 2.2.3. Fragment Analysis

1. Hi-Di™ Formamide (Applied Biosystems, Waltham, MA, USA; Cat. no.: 4440753);
2. POP-7™ Polymer (Applied Biosystems, Waltham, MA, USA; Cat. no.: 4393708);
3. GeneScan™ 500 ROX™ Size Standard (Applied Biosystems, Waltham, MA, USA; Cat. no.: 4393708);
4. DS-30 Matrix Standard kit Dye set D (Applied Biosystems, Waltham, MA, USA; Cat. no.: 4345827);
5. Axygen^®^ 96 well-polypropylene PCR microplate compatible with ABI (Corning Inc., Corning, NY, USA; Cat. no.: PCR-96-AB-C).

### 2.3. Equipment

- Maxwell^®^ RSC Instrument (Promega Corporation, Madison, WI, USA; Cat. no.: AS4500);
- NanoDrop™ 2000 Spectrophotometers (Thermo Fisher Scientific, Waltham, MA, USA; Cat. no.: ND-2000);
- Benchtop vortex mixer;
- Heating block set at 56 °C;
- Rotating tube mixer for liquid blood samples (optional);
- Veriti Thermal Cycler (Applied Biosystems, Waltham, MA, USA; Cat. no.: 4413964);
- 3500 Genetic Analyzer (Applied Biosystems, Waltham, MA, USA; Cat. no.: 4405673);
- Software for fragment analysis.

## 3. Procedure

### 3.1. Genomic DNA (gDNA) Extraction

Fresh or frozen whole blood or bone marrow samples collected in EDTA tubes were used for gDNA extraction using the commercial kit, Maxwell^®^ RSC Blood DNA Kit (Promega Corporation, Madison, WI, USA; Cat. no.: AS1400), and the equipment Maxwell^®^ RSC Instrument (Promega Corporation, Madison, WI, USA; Cat. no.: AS4500). The instructions for use provided by the manufacturer were used. Briefly:

1. Before starting:
  - Heat the heat block to 56 °C for use in step 7;
  - Prepare 1.5 mL eppendorf tubes for each sample for use in step 3.
2. Mix all blood samples by gently inverting 8 to 10 times for at least 5 min at room temperature;
3. Add 300 μL of liquid blood to each 1.5 mL eppendorf tube;
4. Add 300 μL of lysis buffer to each 1.5 mL eppendorf tube;
5. Add 30 μL of proteinase K solution to each 1.5 mL eppendorf tube;
6. Vortex each tube for 10 s;
7. Incubate each tube in the heating block at 56 °C for 20 min.

**OPTIONAL STEP:** during the incubation, the Maxwell^®^ RSC Instrument may be prepared for the run according to the manufacturer’s instructions—select the method (RSC Blood DNA) and the positions that will be used in the extraction run and enter the sample tracking information.

8. After the incubation, transfer all the lysate blood sample to each cartridge into the prober well and place the plunger, as described by the manufacturer;
9. Place an empty elution tube (0.5 mL) into each cartridge at the deck tray;
10. Add 50 μL of Elution Buffer of each elution tube;
11. Insert the deck tray properly into the Maxwell^®^ RSC Instrument;
12. Before starting the extraction run, make sure that all elution tubes are open and all cartridges have a plunger;
13. After checking, the extraction run can begin: touch the Start button. The platform will retract and the door will close. This extraction run takes ≌40 min to finish;
14. After the extraction run has ended, discard all the cartridges and plungers properly and keep with the elution tubes containing the extracted gDNA;
15. Centrifuge the elution tubes at 12,000× *g* for 5 min;
16. In a new 1.5 mL eppendorf tube properly identified, transfer the supernatant.

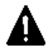
 **CRITICAL STEP:** be careful to transfer the supernatant gently in a way that all the paramagnetic particles are not disturbed after centrifugal separation.

17. Proceed with the quantification of gDNA using the NanoDrop™ 2000 Spectrophotometer (Thermo Fisher Scientific, Waltham, MA, USA; Cat. no.: ND-2000);
18. In a new 1.5 mL eppendorf tube, standardize all the gDNA to 5 ng/μL (A260/A280: 1.7–2.0) and store the pure gDNA at −20 °C.

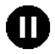
 **PAUSE STEP:** after ending the gDNA quantification, the diluted gDNA can be stored at 4 °C (if the PCR will be performed within 24 h) or −20 °C for long periods.

### 3.2. Amplification of the Target (Conventional PCR)

This step amplifies molecular targets relevant to AML diagnosis and risk stratification [3,11,12]. For the *NPM1* gene, amplification targets exon 12, where the most prevalent mutations (types A, B and D) occur [13]. For internal tandem duplication in the *FLT3* gene, amplification targets exons 14 and 15 [14,15].

1. Before starting:
  - If gDNA samples are stored at −20 °C, they can thaw in the refrigerator (≌4 °C) while the mix is being prepared;
  - Prepare 0.2 mL eppendorf tubes for each gDNA sample for use in step 5;
  - Prepare 1.5 mL eppendorf tube to prepare the mix for use in step 3;
  - The reagents that will be used to prepare the PCR mix (Table 2) must be thawed at room temperature before pipetting (except Taq DNA polymerase: remove from the freezer only when pipetting it).
2. Vortex for 5 s and spin all the components from Table 2 (except Taq DNA polymerase).

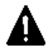
 **CRITICAL STEP:** Taq DNA polymerase must be mixed gently. Pipette “up and down” a few times may be used to mix.

3. Pipette the reagents in a new 1.5 mL eppendorf tube according to the number of gDNA samples for which PCR will be performed. Table 2 contains the components required for the PCR mix as the volumes (μL) for one reaction. Example: if you have three samples, you will need to multiply all the volumes by three.

**OPTIONAL STEP:** It is important to know if the PCR reaction is occurring as desired and to confirm there is no contamination during the process. It is highly recommended to implement a negative and positive control reaction always when a new mix is completed. Water for molecular biology may be used as a negative control and a sample known to be positive for *FLT3*/ITD and *NPM1* mutations as a positive control.

4. Vortex for 5 s and spin the mix prepared;
5. Add 45 μL of the mix in each 0.2 mL eppendorf tube;
6. Add 5 μL of gDNA samples (5 ng/μL) into 0.2 mL eppendorf tubes with the mix, resulting in a final volume of 50 μL.

**OPTIONAL STEP:** the same volume of 5 μL is used for the negative and positive controls.

7. Vortex for 5 s and spin each 0.2 mL eppendorf tube;
8. Place the tubes in the thermocycler with the cycle described (Table 3). This PCR takes ≌100 min to finish.

**Table 3:** PCR cycle used for target amplification.

	Temperature	Time	Cycles
Initial denaturation	95 °C	10 min	1
Denaturation	95 °C	30 s	35
Annealing	55 °C	30 s	
Extension	72 °C	30 s	
Final extension	72 °C	10 min	1
Hold	4 °C		-


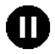
 **PAUSE STEP:** after ending the PCR, the eppendorf tubes containing the PCR product can be stored protected from light at 4 °C (if the PCR will be performed within 24 h) or −20 °C for long periods.

### 3.3. Capillary Electrophoresis

PCR amplification was performed using fluorescently labeled forward primers and unlabeled reverse primers: fluorescein dye (FAM) for *FLT3*/ITD and hexachlorofluorescein dye (HEX) for *NPM1*. In this step, the amplicons will be separated by size using a 3500 Genetic Analyzer (Applied Biosystems, Waltham, MA, USA; Cat. no.: 4405673) with DS-30 Matrix Standard kit dye set D (Applied Biosystems, Waltham, MA, USA; Cat. no.: 4345827) and POP-7™ Polymer (Applied Biosystems, Waltham, MA, USA; Cat. no.: 4393708). GeneScan™ 500 ROX™ Size Standard will be used.

1. Before starting:
  - If PCR products are stored at −20 °C, they can thaw in the refrigerator (≌4 °C) while the mix is being prepared;
  - Prepare the 1.5 mL eppendorf tube to prepare the mix for use in step 3;
  - Prepare the 96-well microplate compatible with the 3500 Genetic Analyzer for use in step 5;
  - Thaw Hi-Di™ Formamide (Applied Biosystems, Waltham, MA, USA; Cat. no.: 4440753) at room temperature before pipetting;
  - **OPTIONAL STEP:** start pre-heating (60 °C) the oven for the 3500 Genetic Analyzer.
2. Briefly vortex and spin the reagents from Table 4;
3. In an empty 1.5 mL eppendorf tube, pipette the reagents from Table 4 according to the number of samples. Example: if you have three samples you will need to multiply all the volumes by three.

4. Vortex for 5 s and spin the mix prepared;
5. In a 96-well microplate, pipette 9.6 μL of the mix in each well for each sample.

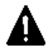
 **CRITICAL STEP:** the 3500 Genetic Analyzer with eight capillaries does the electrophoresis of one column (eight wells) per injection. The pipetting order on the microplate should follow the column order. Example: A1, B1, C1, D1, etc.

6. Add 0.7 μL of the PCR product in the well containing the mix, resulting in a final volume of 10.3 μL per reaction;
7. Pipette “up and down” a few times with a multichannel pipette to homogenize;
8. If the last pipetted column has any empty well, complete it with 10.3 μL of Hi-Di™ Formamide. Every column where electrophoresis will be carried out must not have any empty well.
9. Seal the microplate with septa;
10. Briefly centrifuge the microplate to not leave any contents on the well wall;
11. Incubate the microplate for 3 min at 95 °C to denature the DNA fragments;
12. Immediately place the microplate on ice for ≥2 min.

**OPTIONAL STEP:** during step 12, the set-up of the electrophoresis run may be completed with the 3500 Genetic Analyzer.

13. Briefly centrifuge the microplate;
14. Place the microplate in the 3500 Genetic Analyzer and start the electrophoresis. Information about the electrophoresis instrument protocol to use in this technique is listed in Table 5.

**Table 5:** Parameters to use in capillary electrophoresis.

Parameters	
Application type	Fragment
Capillary Length	50 cm
Polymer	POP 7
Dye Set	D
Oven Temperature	60 °C
Run Time	1330 s
Run Voltage	19.5 kVolts
Pre-Run Time	180 s
Pre-Run Voltage	15 kVolts
Injection Time	8 s
Injection Voltage	1.6 kVolts
Data Delay	1 s
Advanced Options	Default
Sizecaller	SizeCaller v1.1.0

15. When electrophoresis is finished, an FSA file with the electropherogram of each sample will be provided to perform fragment analysis.

## 4. Expected Results

Each sample will generate an electropherogram that can be used for fragment analysis. For the *NPM1* gene, PCR amplification targets a region of exon 12 where the most prevalent mutations occur: type A (c.860_863dupTCTG), type B (c.862_863insCATG) and type D (c.863_864insCCTG), all characterized by the insertion of four base pairs [16]. For the *FLT3* gene, PCR amplification targets exons 14 and 15, where internal tandem duplications (*FLT3*/ITD) occur and may vary in their insertion length [17]. The expected wild-type PCR product sizes are approximately 170 bp for *NPM1* and 329 bp for *FLT3*/ITD.

Furthermore, the fragment peaks are generated by using different dye sets: HEX for the *NPM1* gene (green dye) and FAM for the *FLT3* gene (blue dye).

### 4.1. Size Standard

**Figure 2:**
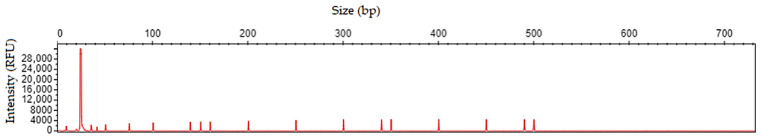
The 500 ROX™ size standard. Dye-labeled fragments ranged from 35–500 bases and their intensity.

The size standard is a fluorescence-based DNA used during the electrophoresis of each sample to precisely determine the DNA fragment size. The 500 ROX™ serves as the size standard, providing 16 single-stranded dye-labeled fragments of 35, 50, 75, 100, 139, 150, 160, 200, 250, 300, 340, 350, 400, 450, 490 and 500 bases. Before analyzing the samples, it is important to analyze if each peak of the size standard is detected correctly (Figure 2).

### 4.2. Sample Without NPM1 and FLT3/ITD Mutations

**Figure 3:**
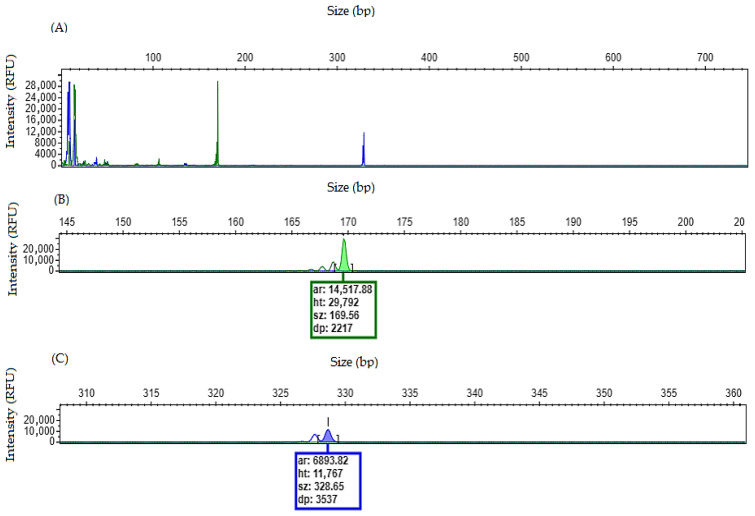
Homozygous wild-type electropherogram for *NPM1* and *FLT3* genes. (**A**) Overview of the electropherogram showing fragment peaks generated when no mutation is detected. (**B**) Zoom-in on the electropherogram showing a single peak, size of 169.56 bp, consistent with wild-type allele for *NPM1* gene. No mutation found. (**C**) Zoom-in on the electropherogram showing a single peak, size of 328.65 bp, consistent with wild-type allele for *FLT3* gene. No mutation found.

When a patient has a homozygous wild-type for both genes, the electropherogram will show a single peak for each gene corresponding to the wild-type allele (Figure 3).

### 4.3. Sample with NPM1 and FLT3/ITD Mutations

**Figure 4:**
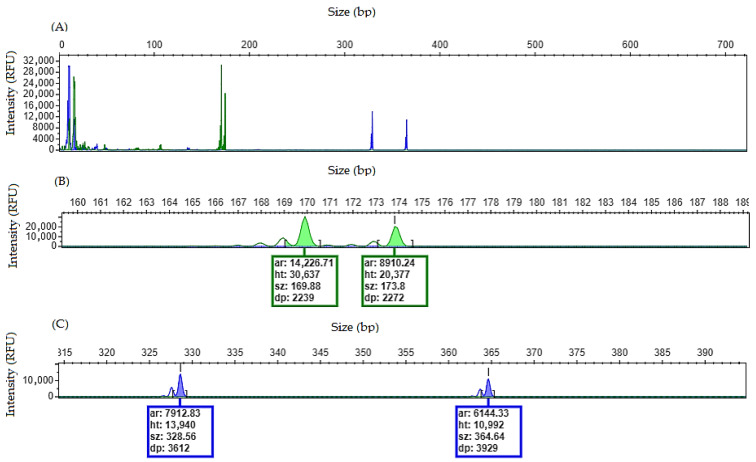
Heterozygous electropherogram for *NPM1* and *FLT3*/ITD genes. (**A**) Overview of the electropherogram showing fragment peaks generated when *NPM1* and *FLT3*/ITD mutations are detected. (**B**) Zoom-in on the electropherogram showing two peaks (difference of ≌4 bp between them), size of 169.88 bp and 173.8 bp, consistent with wild-type and mutant alleles for *NPM1* gene, respectively. (**C**) Zoom-in on the electropherogram showing two peaks, size of 328.56 bp and 364.64 bp, consistent with wild-type and mutant allele for *FLT3* gene, respectively. Internal tandem duplication of ≌36 bp, corresponding to 12 trinucleotide repeats.

When a patient is heterozygous, the electropherogram will show two peaks for each gene: one corresponding to the wild-type allele and one corresponding to the mutant allele (Figure 4). For the *NPM1* gene, exon 12 insertions will generate a fragment that is four base pairs larger than the wild-type amplicon, corresponding to the typical insertion mutations (types A, B and D). *FLT3*/ITD mutations consist of internal tandem duplications whose insertion length may vary between patients, most commonly occurring as in-frame insertions (multiples of three nucleotides). Therefore, the size difference between mutant and wild-type fragments will allow the estimation of the length of the duplicated sequence.

### 4.4. Sample with Only NPM1 Mutation

**Figure 5:**
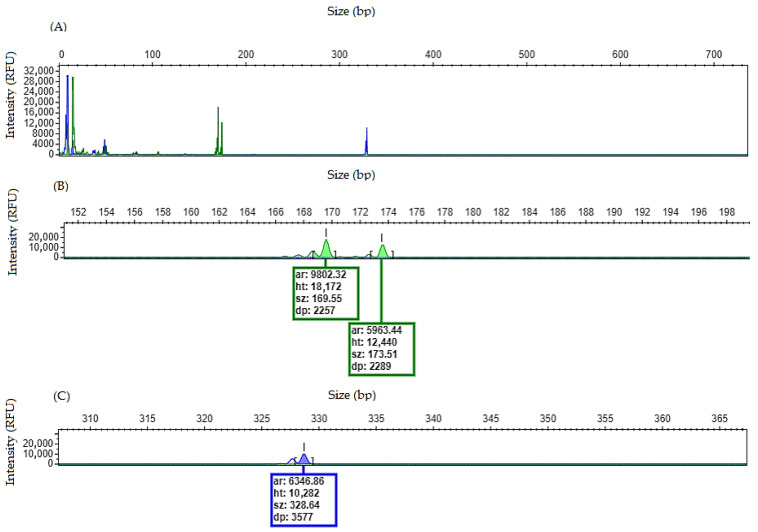
Heterozygous and homozygous wild-type electropherogram for *NPM1* and *FLT3* genes, respectively. (**A**) Overview of the electropherogram showing fragment peaks generated when only an *NPM1* mutation is detected. (**B**) Zoom-in on the electropherogram showing two peaks (difference of ≌4 bp between them), size of 169.55 bp and 173.51 bp, consistent with wild-type and mutant alleles for *NPM1* gene, respectively. (**C**) Zoom-in on the electropherogram showing only one peak, size of 328.64 bp, consistent with wild-type allele for *FLT3* gene. No mutation found.

When a patient is heterozygous for the *NPM1* gene and a homozygous wild-type for the *FLT3* gene, the electropherogram will show two peaks (green dye) corresponding to the wild-type and mutant alleles for *NPM1* and one peak (blue dye) corresponding to the wild-type allele for *FLT3* (Figure 5).

### 4.5. Sample with Only FLT3/ITD Mutation

**Figure 6:**
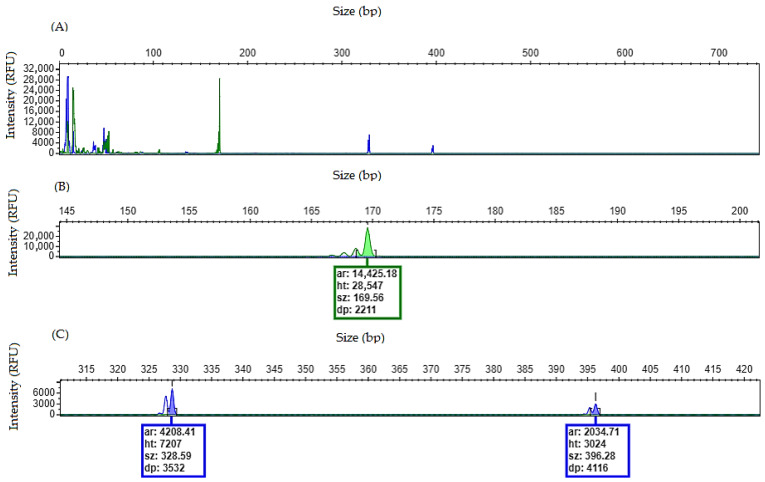
Heterozygous and homozygous wild-type electropherogram for *FLT3* and *NPM1* genes, respectively. (**A**) Overview of the electropherogram showing fragment peaks generated when only a *FLT3*/ITD mutation is detected. (**B**) Zoom-in on the electropherogram showing only one peak, size of 169.56 bp, consistent with wild-type allele for *NPM1* gene. No mutation found. (**C**) Zoom-in on the electropherogram showing two peaks, size of 328.59 bp and 396.28 bp, consistent with wild-type and mutant allele for *FLT3* gene, respectively. Internal tandem duplication of ≌68 bp.

An electropherogram for a patient heterozygous for the *FLT3*/ITD and homozygous for the *NPM1* gene will have two peaks (blue dye) corresponding to the wild-type and mutant alleles for *FLT3* and one peak (green dye) corresponding to the wild-type allele for *NPM1* (Figure 6). Remember, the size difference between the mutant and wild-type fragments will allow the estimation of the duplicated sequence length for the *FLT3*/ITD mutations.

### 4.6. FLT3/ITD Allelic Burden Calculation

Although the allelic ratio (AR) of *FLT3*/ITD is no longer considered in the risk stratification [12], it may still be useful for confirming *FLT3*/ITD positivity (cut off AR ≥ 0.05) [13,18], particularly when low-intensity peaks are observed.

The AR may be calculated using the peak height:

Allelic burden = ∑height of mutant peak(s)/height of wild-type peak

Alternatively, the peak area may be used:

Allelic burden = ∑area of mutant peak(s)/area of wild-type peak

**Figure 7:**
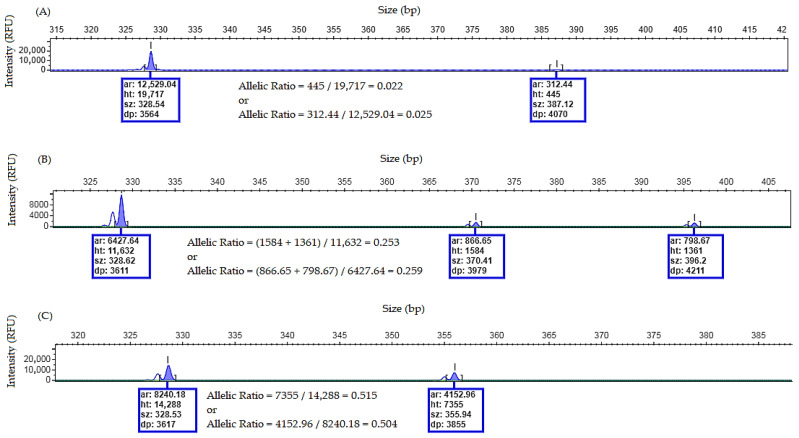
Three examples of allelic ratio calculation for *FLT3*/ITD by using height (ht) and area (ar) of the peaks. (**A**) Electropherogram showing an allelic ratio of ≌0.02 (≤cut off of 0.05). Based on allelic ratio, this sample is negative for *FLT3*/ITD. (**B**) Electropherogram showing an example of a positive sample for *FLT3*/ITD with a low allelic ratio ≌ 0.2 with two mutant peaks. (**C**) Electropherogram showing an example of a positive sample for *FLT3*/ITD with a high allelic ratio ≌ 0.5.

See the examples below (Figure 7):

When off-scale peaks are present, an allelic ratio calculation is not reliable because fluorescence signal saturation results in an inaccurate peak height and area measurements. In such cases, the PCR product should be diluted before reinjection or the amount of input DNA in the PCR reaction should be reduced to obtain measurements within the linear detection range.

### 4.7. Quality Control

Quality control measures are essential to ensure the reliability of both the experimental procedure and fragment analysis interpretation. The main quality assurance considerations discussed throughout the protocol are summarized below as a practical checklist for routine use.

Before performing the assay:

- Confirm that the maintenance and calibration of all equipment are up to date.
- Verify that all reagents are properly stored and within their expiration dates.
- Ensure that genomic DNA samples are properly quantified and standardized.

Before patient’s electropherogram interpretation:

- Verify the correct detection of the size standard peaks (see Section 4.1). The size standard included in each sample allows the software to generate a sample-specific sizing curve, enabling accurate fragment size determination by comparing migration patterns during electrophoresis.
  ○ Confirm that the size standard generated a consistent sizing curve for the sample.
  ○ Ensure fragment sizing is consistent across the expected range.
- Control samples evaluation:
  ○ Negative controls should display only the wild-type fragment size (approximately 170 bp for *NPM1* and 329 bp for *FLT3*/ITD).
    ▪ The absence of amplification may indicate PCR failure. If observed, repeat PCR and fragment analysis.
    ▪ Unexpected peaks may indicate contamination. If observed, repeat PCR and fragment analysis.
  ○ Positive controls must reproduce previously characterized mutation patterns. Should display the wild-type and mutant fragment sizes.
    ▪ *NPM1* mutant allele should appear 4 bp larger than the wild-type. If not observed, repeat PCR and fragment analysis.
    ▪ *FLT3*/ITD fragment size depends on the known insertion length of the control. If not observed, repeat PCR and fragment analysis.
  ○ No template control (NTC): must not show any amplification peaks. Otherwise, the PCR and fragment analysis must be repeated due to the possible presence of contamination.

During electropherogram interpretation:

- Occasionally, smaller peaks detected before the wild-type fragment may be observed during fragment analysis.
  ○ These may correspond to small deletions, sequence variants or PCR-related artifacts within the amplified region and should not be misinterpreted as insertion-type mutations.
    ▪ When such patterns are observed, interpretation should be performed cautiously in the context of the electropherogram profile and clinical laboratory findings. If necessary, repeat amplification or complementary molecular methods may be considered.
- Off-scale peaks may occur due to fluorescence signal saturation. As this assay is interpreted qualitatively (the presence or absence of mutations), off-scale peaks do not typically compromise mutation detection or interpretation. However:
  ○ The repeated injection of saturated signals may negatively affect the long-term instrument performance.
    ▪ The dilution of PCR products before reinjection is recommended as good laboratory practice.
  ○ When AR is required, off-scale peaks must not be used:
    ▪ The PCR product should be diluted before reinjection, or the sample input in PCR should be reduced to obtain measurements within the linear detection range.

As this protocol describes an in-house assay, laboratories implementing the method should incorporate validation procedures into their local quality management systems, in accordance with institutional and accreditation requirements.

## 5. Conclusions

The protocol described here for detecting *NPM1* mutations and *FLT3*/ITD for use in AML diagnosis is simple, easy, fast and inexpensive to implement in a laboratory when compared to other molecular techniques. Furthermore, using the same analysis for both mutations, it was possible to optimize this assay and laboratory routine with a multiplex reaction.

## Figures and Tables

**Table 1:** Primers sequences used in PCR for detection of *NPM1* mutations and Fms-like tyrosine kinase 3 internal tandem duplication (*FLT3*/ITD).

	Sequence (5′ → 3′)	PCR Product Size (bp)
*NPM1* Forward	ATTTCTTTTTTTTTTTTTCCAGGCTATTCAAG	
*NPM1* Reverse	HEX—CACGGTAGGGAAAGTTCTCACTCTGC	172
*FLT3*/ITD Forward	GCAATTTAGGTATGAAAGCCAGC	
*FLT3*/ITD Reverse	FAM—CTTTCAGCATTTTGACGGCAACC	329

**Table 2:** Components required for the PCR mix, respective volumes and the final concentration.

Components	Volume (μL)/Reaction	Final Concentration
10× Buffer	5	1×
MgCl_2_ 50 mM	1.5	1.5 mM
dNTP mix 2.5 mM	4	0.2 mM
*NPM1* Primer Forward 10 μM	1	0.2 μM
*NPM1* Primer Reverse 10 μM	1	0.2 μM
*FLT3*/ITD Primer Forward 10 μM	1	0.2 μM
*FLT3*/ITD Primer Reverse 10 μM	1	0.2 μM
Taq DNA Polymerase (5 U/μL)	0.5	250 U
Nuclease free-water	30	-
Total	45	-

**Table 4:** Reagents for capillary electrophoresis and the volume required for one reaction.

Components	Volume (μL)/Reaction
500 ROX™ Size Standard	0.3
Hi-Di™ Formamide	9.3
Total	9.6

## Data Availability

The data presented in this study are available on request from the corresponding author.

## Supplementary Materials

The following supporting information can be downloaded at: https://www.mdpi.com/article/10.3390/mps9020059/s1, Table S1: Comparison of results obtained using the multiplex assay and the singleplex reference test for *NPM1* mutation detection, including concordance percentage. Table S2: Comparison between multiplex assay results and the singleplex reference test for *FLT3*/ITD mutation detection, including concordance percentage and mean standard deviation (SD) of signal ratios obtained in each assay. Table S3: Duplicate analysis results used for concordance assessment of the multiplex PCR–capillary electrophoresis assay (highlighted in pink) compared with the singleplex reference assay (highlighted in orange) for *FLT3*/ITD mutation detection.; Table S4: Results obtained for concordance assessment during validation of the multiplex PCR with capillary electrophoresis (highlighted in pink), compared with the reference test (singleplex PCR with capillary electrophoresis, highlighted in orange) for *NPM1* mutation detection. Table S5: Results obtained in intra-assay and inter-assay analyses for concordance assessment of the multiplex PCR–capillary electrophoresis assay in the detection of *FLT3*/ITD and *NPM1* mutations for precision performance evaluation. Table S6: Results obtained in intra-assay and inter-assay analyses for concordance assessment of the multiplex PCR–capillary electrophoresis assay in detection of *FLT3*/ITD and *NPM1* mutations for precision performance evaluation. Table S7: General information on the primers used in the multiplex assay for detection of *FLT3*/ITD and *NPM1* mutations. Table S8: Most prevalent *NPM1* gene mutations, corresponding nucleotide insertions and GenBank accession numbers of the reference sequences (RefSeq). Figure S1: In silico primer specificity analysis for *NPM1* mutation detection (RefSeq NG_016018.1), targeting exon 11, performed using NCBI Primer-BLAST. Figure S2: In silico primer specificity analysis for *FLT3*/ITD mutation detection (RefSeq NG_007066.1), targeting exons 14 and 15, performed using NCBI Primer-BLAST. Figure S3: (A) Nucleotide sequence of the amplified fragment obtained from alignment of the primer pair targeting the *NPM1* gene. (B) Nucleotide sequence of the amplified fragment obtained from alignment of the primer pair targeting the *FLT3* gene. Generated using NCBI GenBank. Figure S4: (A) General alignment information comparing the amplified fragment (query) with different *NPM1* mutation sequences (subject). (B) Alignment of the amplified fragment with the wild-type *NPM1* sequence, showing 100% identity. (C) Alignment of the amplified fragment with a type A *NPM1* mutation sequence, showing 98% identity, with the remaining 2% corresponding to the insertion gap of four base pairs (TCTG). (D) Alignment of the amplified fragment with a type B *NPM1* mutation sequence, showing 98% identity, with the remaining 2% corresponding to the insertion gap of four base pairs (CATG). (E) Alignment of the amplified fragment with a type D *NPM1* mutation sequence, showing 98% identity, with the remaining 2% corresponding to the insertion gap of four base pairs (CCTG). Figure S5: (A) General alignment information of the amplified fragment (query) with the sequence showing the highest identity percentage. (B) Alignment of the amplified fragment generated using the primer pair targeting exons 14 and 15 of the *FLT3* gene with the wild-type sequence, showing 100% identity.
