# Supplementary material for: A Simple and Fast Protocol to Detect Nucleophosmin 1 (NPM1) Mutation and Fms-like Tyrosine Kinase 3 Internal Tandem Duplication (FLT3/ITD): Optimizing Laboratory Routine
Source: Methods Protoc. 2026 Apr 3;9(2):59. doi: 10.3390/mps9020059 (PMC13119083; doi:10.3390/mps9020059)
Supplement: Supplementary file 1 [file mps-09-00059-s001.zip › mps-4076543-supplementary.pdf]

Supplementary Material

For the implementation of a diagnostic assay in routine clinical laboratory practice, it is necessary to validate the analytical performance of the method to ensure reliability of results, minimize errors and prevent potential impact on patient care. The validation report for detection of the *FLT3*/ITD and *NPM1* mutations via multiplex PCR and capillary electrophoresis is presented below and organized into the following sections: general information, data analysis and conclusion.

1. General Information

1.1 Method Description

We utilized an in vitro nucleic acid amplification assay based on conventional multiplex polymerase chain reaction (PCR), followed by capillary electrophoresis for fragment analysis, to support qualitative detection of the *FLT3*/ITD and *NPM1* mutations in whole blood or bone marrow samples collected in EDTA tubes.

1.2 Reagents and Manufacturers

- Taq DNA Polymerase (500 U), 10× buffer and $MgCl_2$ (5 U/µL)—Ludwig Biotecnologia (Cat. no. 35);
- Primers for *NPM1* (10 µM)—Applied Biosystems

Forward: 5′-ATT TCT TTT TTT TTT TTT CCA GGC TAT TCA AG-3′
Reverse: 5′-HEX-CAC GGT AGG GAA AGT TCT CAC TCT GC-3′

- Primers for *FLT3*/ITD (10 µM)—Sigma-Aldrich

Forward: 5′-GCA ATT TAG GTA TGA AAG CCA GC-3′
Reverse: 5′-FAM-CTT TCA GCA TTT TGA CGG CAA CC-3′

- dNTP set (2.5 mM each: dATP, dCTP, dGTP, dTTP)—Quatro G Biotecnologia (Cat. no. 100018).

1.3 Performance Tests

- In silico specificity;
- Concordance (veracity);
- Precision (repeatability/reproducibility).

1.4 Reference Tests

Conventional PCR followed by capillary electrophoresis for mutation detection in:

- *FLT3*/ITD gene (singleplex assay);
- *NPM1* gene (singleplex assay).

1.5 Number of Tests

A total of 40 tests were performed.

1.6 Sample Origin

Samples included were obtained from routine clinical laboratory specimens, synthetic genetic fragments and external quality assessment materials (UK NEQAS).

1.7 Sample Distribution

A total of 40 samples were included:

- Eight samples without *FLT3*/ITD or *NPM1* mutations;
- Ten samples with both *FLT3*/ITD and *NPM1* mutations;
- Nine samples with *NPM1* mutation only.
- Thirteen samples with *FLT3*/ITD mutation only.

1.8 Sample Matrix

- Twenty-three bone marrow samples (EDTA);
- Seven whole blood samples (EDTA);
- Eight synthetic samples from external quality assessment (UK NEQAS);

- One synthetic *FLT3*/ITD mutant fragment.
- One synthetic *NPM1* mutant fragment.

2. Data analysis

2.1 Performance Evaluation: Concordance (Veracity)

- **Test:** Concordance percentage;
- **Result:** 100% concordance;
- **Comments and interpretation:**

A 100% concordance rate was observed for mutation detection in both the *NPM1* (Table S1) and *FLT3*/ITD genes (Table S2) when the multiplex reaction was used, as opposed to the use of singleplex assays (perfect agreement; Cohen's kappa = 1). Among the 40 samples tested, 22 samples showed *FLT3*/ITD mutations detected in duplicate analyses, allowing signal ratio calculation (Table S3). Of these, four samples showed discordant allelic burden classification (high vs. low) between the singleplex and multiplex reactions. However, in all discordant cases, the signal ratio values were close to the cutoff value (0.5), which may explain the classification differences, particularly considering the low standard deviation observed among replicate measurements (Table S2). For *NPM1* mutation detection, no discrepancies were observed between multiplex and singleplex assays (Table S4).

Table S1. Comparison of results obtained using the multiplex assay and the singleplex reference test for *NPM1* mutation detection, including concordance percentage.

		***NPM1* multiplex**	
		**Detected**	**Not detected**
***NPM1* singleplex**	**Detected**	19	0
	**Not detected**	0	21
	**Total samples**	40	
	**Concordant samples**	40	
	**Concordance percentage**	100%	
	**Kappa value**	1	

Table S2. Comparison between multiplex assay results and the singleplex reference test for *FLT3*/ITD mutation detection, including concordance percentage and mean standard deviation (SD) of signal ratios obtained in each assay.

		***FLT3*/ITD multiplex**		**Mean SD of signal ratios**
		**Detected**	**Not detected**	
***FLT3*/ITD singleplex**	**Detected**	23	0	0.174
	**Not detected**	0	17	
	**Total samples**	40		
	**Concordant samples**	40		
	**Concordance percentage**	100%		
	**Kappa value**	1		
	**Mean SD of signal ratios**	0.059		

Table S3. Duplicate analysis results used for concordance assessment of the multiplex PCR–capillary electrophoresis assay (highlighted in pink) compared with the singleplex reference assay (highlighted in orange) for *FLT3*/ITD mutation detection.

Sample	*FLT3*/ITD singleplex (detected if SR ≥ 0.05)	WT Allele Size	WT Allele Area	Mutant Allele 1—Size	Mutant Allele 1—Area	Mutant Allele 2—Size	Mutant Allele 2—Area	SR	Allelic Burden (high if SR ≥ 0.5)	SR Mean and Allelic Burden	*FLT3*/ITD multiplex (detected if SR ≥ 0.05)	WT Allele Size	WT Allele Area	Mutant Allele 1—Size	Mutant Allele 1—Area	Mutant Allele 2—Size	Mutant Allele 2—Area	SR	Allelic Burden (high if SR ≥ 0.5)	SR Mean and Allelic Burden	Concord.
1	NOT DETECTED	328.41	21569.25	-	-	-	-	-	-	-	NOT DETECTED	328.73	16836.2	-	-	-	-	-	-	-	Yes
	NOT DETECTED	328.54	12529.04	387.12	312.44	-	-	0.02	-		NOT DETECTED	328.64	6346.86	-	-	-	-	-	-		
2	NOT DETECTED	328.33	13979.64	-	-	-	-	-	-	-	NOT DETECTED	328.69	12855.36	-	-	-	-	-	-	-	Yes
	NOT DETECTED	328.61	19706.2	-	-	-	-	-	-		NOT DETECTED	328.71	10799.16	-	-	-	-	-	-		
3	DETECTED	328.21	11819.40	384.54	8090.47	-	-	0.68	HIGH	0.64	DETECTED	328.73	6326.14	384.37	3111.25	-	-	0.49	LOW	0.51	Yes
	DETECTED	328.7	14110.83	384.3	8270.48	-	-	0.59	HIGH	HIGH	DETECTED	328.66	4074.63	384.62	2148.64	-	-	0.53	HIGH	HIGH	
4	NOT DETECTED	328.30	7260.55	-	-	-	-	-	-	-	NOT DETECTED	328.69	8019.17	-	-	-	-	-	-	-	Yes
	NOT DETECTED	328.79	12731.48	-	-	-	-	-	-		NOT DETECTED	328.73	8515.88	-	-	-	-	-	-		
5	DETECTED	328.34	8511.54	393.10	20218.95	-	-	2.38	HIGH	2.20	DETECTED	328.85	3772.72	393.45	6874.47	-	-	1.82	HIGH	1.75	Yes
	DETECTED	328.61	7757.61	393.08	15780.73	-	-	2.03	HIGH	HIGH	DETECTED	328.71	2337.38	393.35	3934.47	-	-	1.68	HIGH	HIGH	
6	DETECTED	328.59	1736.79	352.56	271.26	-	-	0.16	LOW	0.20	DETECTED	328.78	8547.82	353.03	1221.99	-	-	0.14	LOW	0.14	Yes
	DETECTED	328.64	3955.19	352.71	958.19	-	-	0.24	LOW	LOW	DETECTED	328.55	7188.16	352.82	1003.25	-	-	0.14	LOW	LOW	
7	DETECTED	328.15	25299.98	352.40	1766.39	-	-	0.07	LOW	0.09	DETECTED	328.76	20347.38	352.82	1438.57	-	-	0.07	LOW	0.06	Yes
	DETECTED	328.7	6180.87	352.50	638.66	-	-	0.10	LOW	LOW	DETECTED	328.65	7969.54	352.72	459.77	-	-	0.06	LOW	LOW	
8	NOT DETECTED	328.29	23547.65	-	-	-	-	-	-	-	NOT DETECTED	328.68	14969.34	-	-	-	-	-	-	-	Yes
	NOT DETECTED	328.44	11893.55	-	-	-	-	-	-		NOT DETECTED	328.57	8896.77	-	-	-	-	-	-		
9	NOT DETECTED	328.20	22473.63	-	-	-	-	-	-	-	NOT DETECTED	328.62	11863.3	-	-	-	-	-	-	-	Yes
	NOT DETECTED	328.53	5727.83	-	-	-	-	-	-		NOT DETECTED	328.6	13002.17	-	-	-	-	-	-		
10	NOT DETECTED	328.30	21690.48	-	-	-	-	-	-	-	NOT DETECTED	328.68	12774.06	-	-	-	-	-	-	-	Yes
	NOT DETECTED	328.53	16981.81	-	-	-	-	-	-		NOT DETECTED	328.62	11990.02	-	-	-	-	-	-		
11	DETECTED	328.33	44127.74	352.6	7191.37	420.30	4583.4	0.27	LOW	0.19	DETECTED	328.7	11640.75	352.88	1056.11	420.66	505.83	0.13	LOW	0.12	Yes
	DETECTED	328.51	13691.67	352.55	910.14	420.39	719.7	0.12	LOW	LOW	DETECTED	328.66	8635.54	352.85	553.52	420.74	303.28	0.10	LOW	LOW	
12*	NOT DETECTED	-	-	-	-	-	-	-	-	-	NOT DETECTED	-	-	-	-	-	-	-	-	-	Yes
	NOT DETECTED	-	-	-	-	-	-	-	-		NOT DETECTED	-	-	-	-	-	-	-	-		

Sample	*FLT3*/ITD singleplex (detected if SR ≥ 0.05)	WT Allele Size	WT Allele Area	Mutant Allele 1—Size	Mutant Allele 1—Area	Mutant Allele 2—Size	Mutant Allele 2—Area	SR	Allelic Burden (high if SR ≥ 0.5)	SR Mean and Allelic Burden	*FLT3*/ITD multiplex (detected if SR ≥ 0.05)	WT Allele Size	WT Allele Area	Mutant Allele 1—Size	Mutant Allele 1—Area	Mutant Allele 2—Size	Mutant Allele 2—Area	SR	Allelic Burden (high if SR ≥ 0.5)	SR Mean and Allelic Burden	Concord.
13	DETECTED	328.25	17090.11	355.44	10066.22	-	-	0.59	HIGH	0.64	DETECTED	328.53	8240.18	355.94	4152.96	-	-	0.50	HIGH	0.52	Yes
	DETECTED	328.38	9956.42	355.58	6856.95	-	-	0.69	HIGH	HIGH	DETECTED	328.56	5082.66	355.67	2681.06	-	-	0.53	HIGH	HIGH	
14	DETECTED	328.18	12918.41	396.02	6468.48	-	-	0.50	HIGH	0.32	DETECTED	328.59	6830.35	396.31	2144.40	-	-	0.31	LOW	0.28	Yes
	DETECTED	328.53	9334	395.93	1307.03	-	-	0.14	LOW	LOW	DETECTED	328.59	3355.07	396.17	805.36	-	-	0.24	LOW	LOW	
15	DETECTED	328.22	17462.71	369.93	2331.21	395.78	4179.7	0.37	LOW	0.41	DETECTED	328.62	6427.64	370.41	866.65	396.2	798.67	0.26	LOW	0.23	Yes
	DETECTED	328.51	16522.82	369.99	4119.69	395.89	3432.3	0.46	LOW	LOW	DETECTED	328.6	4330.01	370.11	456.15	396.01	375.45	0.19	LOW	LOW	
16	DETECTED	328.20	11993.98	387.19	8268.85	-	-	0.69	HIGH	0.66	DETECTED	328.58	10768.61	387.57	4346.71	-	-	0.40	LOW	0.36	Yes
	DETECTED	328.44	19280.53	387.16	11972.58	-	-	0.62	HIGH	HIGH	DETECTED	328.65	3134.79	387.48	990.64	-	-	0.32	LOW	LOW	
17	DETECTED	328.32	8904.72	375.72	6143.06	-	-	0.69	HIGH	0.68	DETECTED	328.74	7297.69	375.99	4634.08	-	-	0.64	HIGH	0.61	Yes
	DETECTED	328.47	15909.21	375.66	10730.5	-	-	0.67	HIGH	HIGH	DETECTED	328.61	5133.5	375.9	3018.33	-	-	0.59	HIGH	HIGH	
18	DETECTED	328.20	20003.11	358.31	11061.44	-	-	0.55	HIGH	0.55	DETECTED	328.53	10301.50	358.61	4504.58	-	-	0.44	LOW	0.44	Yes
	DETECTED	328.18	19210.38	358.29	10583.68	-	-	0.55	HIGH	HIGH	DETECTED	328.67	6774.37	358.5	3027.71	-	-	0.45	LOW	LOW	
19	DETECTED	328.24	25399.16	381.63	2640.68	-	-	0.10	LOW	0.09	DETECTED	328.57	13937.23	382.03	780.19	-	-	0.06	LOW	0.05	Yes
	DETECTED	328.33	21676.28	381.59	1523.66	-	-	0.07	LOW	LOW	DETECTED	328.59	7143.37	381.77	375.85	-	-	0.05	LOW	LOW	
20	DETECTED	328.11	15930.98	364.27	13797.65	-	-	0.87	HIGH	1.00	DETECTED	328.56	7912.83	364.64	6144.33	-	-	0.78	HIGH	0.77	Yes
	DETECTED	328.52	5674.35	364.12	6474.24	-	-	1.14	HIGH	HIGH	DETECTED	328.68	3891.66	364.5	3003.05	-	-	0.77	HIGH	HIGH	
21	DETECTED	328.15	13389.22	346.64	8802.61	-	-	0.66	HIGH	0.71	DETECTED	328.5	6108.19	347.02	4103.69	-	-	0.67	HIGH	0.69	Yes
	DETECTED	328.45	12647.06	346.76	9719.38	-	-	0.77	HIGH	HIGH	DETECTED	328.49	4600.06	347.07	3271.12	-	-	0.71	HIGH	HIGH	
22	DETECTED	328.05	15048.44	358.24	10228.54	-	-	0.68	HIGH	0.72	DETECTED	328.53	7765.24	358.59	3804.15	-	-	0.49	LOW	0.48	Yes
	DETECTED	328.51	3751.44	358.13	2889.18	-	-	0.77	HIGH	HIGH	DETECTED	328.49	6502.29	358.53	3091.03	-	-	0.48	LOW	LOW	
23	DETECTED	328.04	915.07	393.00	6428.07	-	-	7.02	HIGH	7.98	DETECTED	328.62	1613.68	393.14	10431.25	-	-	6.46	HIGH	6.08	Yes
	DETECTED	328.45	2170.13	392.81	19407.44	-	-	8.94	HIGH	HIGH	DETECTED	328.5	999.49	393.11	5702.65	-	-	5.71	HIGH	HIGH	
24	DETECTED	328.11	13951.25	396.05	9824.05	-	-	0.70	HIGH	0.61	DETECTED	328.59	9152.08	396.41	4118.18	-	-	0.45	LOW	0.45	Yes
	DETECTED	328.38	20068.44	396.03	10342.06	-	-	0.52	HIGH	HIGH	DETECTED	328.56	5699.03	396.28	2544.36	-	-	0.45	LOW	LOW	
25	DETECTED	328.22	16693.31	358.27	1412.54	-	-	0.08	LOW	0.08	DETECTED	328.5	11388.98	358.5	682.59	-	-	0.06	LOW	0.06	Yes
	DETECTED	328.33	24091.65	358.16	1916.09	-	-	0.08	LOW	LOW	DETECTED	328.49	11435.42	358.43	706.4	-	-	0.06	LOW	LOW	
26	DETECTED	328.20	16577.57	358.42	1247.35	453.74	1769.2	0.18	LOW	0.20	DETECTED	328.53	15447.35	358.73	1047.5	454.11	837.18	0.12	LOW	0.12	Yes

Sample	*FLT3*/ITD singleplex (detected if SR ≥ 0.05)	WT Allele Size	WT Allele Area	Mutant Allele 1—Size	Mutant Allele 1—Area	Mutant Allele 2—Size	Mutant Allele 2—Area	SR	Allelic Burden (high if SR ≥ 0.5)	SR Mean and Allelic Burden	*FLT3*/ITD multiplex (detected if SR ≥ 0.05)	WT Allele Size	WT Allele Area	Mutant Allele 1—Size	Mutant Allele 1—Area	Mutant Allele 2—Size	Mutant Allele 2—Area	SR	Allelic Burden (high if SR ≥ 0.5)	SR Mean and Allelic Burden	Concord.
	DETECTED	328.45	19116.16	358.39	2716.35	453.72	1284.4	0.21	LOW	LOW	DETECTED	328.55	12362.53	358.61	867.8	454.04	655.61	0.12	LOW	LOW	
27	DETECTED	328.18	19086.70	453.82	3744.52	-	-	0.20	LOW	0.25	DETECTED	328.57	15302.56	454.14	2044.99	-	-	0.13	LOW	0.12	Yes
	DETECTED	328.42	18921.71	453.73	5690.22	-	-	0.30	LOW	LOW	DETECTED	328.56	9821.95	454.07	1044.72	-	-	0.11	LOW	LOW	
28	DETECTED	328.27	23145.71	358.42	5133.40	-	-	0.22	LOW	0.26	DETECTED	328.44	10115.97	358.7	1803.41	-	-	0.18	LOW	0.17	Yes
	DETECTED	328.39	19506.95	358.24	5950.44	-	-	0.31	LOW	LOW	DETECTED	328.49	6439.48	358.6	1025.01	-	-	0.16	LOW	LOW	
29	NOT DETECTED	328.35	34495.22	-	-	-	-	-	-	-	NOT DETECTED	328.53	11192.76	-	-	-	-	-	-	-	Yes
	NOT DETECTED	328.29	22193.02	-	-	-	-	-	-		NOT DETECTED	328.56	7396.65	-	-	-	-	-	-		
30	DETECTED	328.18	4629.77	358.32	20821.57	-	-	4.50	HIGH	3.88	DETECTED	328.53	2061.05	358.62	6498.15	-	-	3.15	HIGH	2.95	Yes
	DETECTED	328.33	4940.25	358.39	16129.75	-	-	3.26	HIGH	HIGH	DETECTED	328.65	1439.15	358.59	3941.65	-	-	2.74	HIGH	HIGH	
31**	DETECTED	-	-	350.79	53101.84	-	-	-	-	-	DETECTED	-	-	351.55	33015.99	-	-	-	-	-	Yes
	DETECTED	-	-	351.01	43669.87	-	-	-	-		DETECTED	-	-	351.25	25069.3	-	-	-	-		
32	NOT DETECTED	328.27	17099.61	-	-	-	-	-	-	-	NOT DETECTED	328.56	16944.26	-	-	-	-	-	-	-	Yes
	NOT DETECTED	328.23	23273.78	-	-	-	-	-	-		NOT DETECTED	328.58	6344.4	-	-	-	-	-	-		
33	NOT DETECTED	328.14	24505.89	-	-	-	-	-	-	-	NOT DETECTED	328.47	18660.43	-	-	-	-	-	-	-	Yes
	NOT DETECTED	328.2	23765.44	-	-	-	-	-	-		NOT DETECTED	328.62	15985.79	-	-	-	-	-	-		
34	NOT DETECTED	328.11	25128.55	-	-	-	-	-	-	-	NOT DETECTED	328.53	14179.29	-	-	-	-	-	-	-	Yes
	NOT DETECTED	328.33	20155.75	-	-	-	-	-	-		NOT DETECTED	328.65	11773.1	-	-	-	-	-	-		
35	NOT DETECTED	327.88	47228.25	-	-	-	-	-	-	-	NOT DETECTED	328.45	8062.27	-	-	-	-	-	-	-	Yes
	NOT DETECTED	328.05	36648.94	-	-	-	-	-	-		NOT DETECTED	328.56	3433.98	-	-	-	-	-	-		
36	NOT DETECTED	328.06	24487.43	-	-	-	-	-	-	-	NOT DETECTED	328.56	10295.86	-	-	-	-	-	-	-	Yes
	NOT DETECTED	328.24	27256.14	-	-	-	-	-	-		NOT DETECTED	328.58	9237.09	-	-	-	-	-	-		
37	NOT DETECTED	327.97	28456.65	-	-	-	-	-	-	-	NOT DETECTED	328.54	9441.42	-	-	-	-	-	-	-	Yes
	NOT DETECTED	328.29	13419.07	-	-	-	-	-	-		NOT DETECTED	328.52	7509.96	-	-	-	-	-	-		
38	NOT DETECTED	328.06	23894.19	-	-	-	-	-	-	-	NOT DETECTED	328.53	9254.16	-	-	-	-	-	-	-	Yes
	NOT DETECTED	328.21	25726.32	-	-	-	-	-	-		NOT DETECTED	328.52	6971.98	-	-	-	-	-	-		
39	NOT DETECTED	328.18	14286.13	-	-	-	-	-	-	-	NOT DETECTED	328.51	6131.19	-	-	-	-	-	-	-	Yes
	NOT DETECTED	328.3	18844.52	-	-	-	-	-	-		NOT DETECTED	328.92	9071.06	-	-	-	-	-	-		

Sample	*FLT3*/ITD singleplex (detected if SR ≥ 0.05)	WT Allele Size	WT Allele Area	Mutant Allele 1—Size	Mutant Allele 1—Area	Mutant Allele 2—Size	Mutant Allele 2—Area	SR	Allelic Burden (high if SR ≥ 0.5)	SR Mean and Allelic Burden	*FLT3*/ITD multiplex (detected if SR ≥ 0.05)	WT Allele Size	WT Allele Area	Mutant Allele 1—Size	Mutant Allele 1—Area	Mutant Allele 2—Size	Mutant Allele 2—Area	SR	Allelic Burden (high if SR ≥ 0.5)	SR Mean and Allelic Burden	Concord.
40	NOT DETECTED	328.18	23380.21	-	-	-	-	-	-	-	NOT DETECTED	328.53	12361.92	-	-	-	-	-	-	-	Yes
	NOT DETECTED	328.24	27764.26	-	-	-	-	-	-		NOT DETECTED	328.58	11187.38	-	-	-	-	-	-		

Abbreviations: WT, wild-type; * synthetic *NPM1* mutant fragment (no WT allele); ** synthetic *FLT3*/ITD mutant fragment (no WT allele); SR, signal ratio; Concord., concordance.

Table S4. Results obtained for concordance assessment during validation of the multiplex PCR with capillary electrophoresis (highlighted in pink), compared with the reference test (singleplex PCR with capillary electrophoresis, highlighted in orange) for *NPM1* mutation detection.

Sample	*NPM1* singleplex	WT Allele Size	Mutant Allele Size	*NPM1* multiplex	WT Allele Size	Mutant Allele Size	Concordance
1	DETECTED	169.76	173.66	DETECTED	169.81	173.74	Yes
2	DETECTED	169.98	173.94	DETECTED	169.81	173.81	Yes
3	DETECTED	169.78	173.67	DETECTED	169.77	173.66	Yes
4	DETECTED	169.70	173.73	DETECTED	169.93	173.81	Yes
5	DETECTED	169.80	173.71	DETECTED	169.89	173.92	Yes
6	DETECTED	169.62	173.76	DETECTED	169.98	174.01	Yes
7	DETECTED	169.73	176.65	DETECTED	170.01	173.92	Yes
8	DETECTED	169.80	173.70	DETECTED	170.06	173.94	Yes
9	DETECTED	169.81	173.71	DETECTED	169.83	173.78	Yes
10	DETECTED	169.93	173.92	DETECTED	169.85	173.88	Yes
11	DETECTED	169.95	173.94	DETECTED	169.79	173.76	Yes
12*	DETECTED	-	173.40	DETECTED	-	174.12	Yes
13	DETECTED	169.64	173.66	DETECTED	169.88	173.8	Yes

Sample	*NPM1* singleplex	WT Allele Size	Mutant Allele Size	*NPM1* multiplex	WT Allele Size	Mutant Allele Size	Concordance
14	NOT DETECTED	169.70	-	NOT DETECTED	169.92	-	Yes
15	NOT DETECTED	169.70	-	NOT DETECTED	169.91	-	Yes
16	NOT DETECTED	169.70	-	NOT DETECTED	169.74	-	Yes
17	NOT DETECTED	169.73	-	NOT DETECTED	169.81	-	Yes
18	NOT DETECTED	169.82	-	NOT DETECTED	170.00	-	Yes
19	NOT DETECTED	169.68	-	NOT DETECTED	169.88	-	Yes
20	DETECTED	169.80	173.70	DETECTED	169.88	173.8	Yes
21	NOT DETECTED	169.80	-	NOT DETECTED	169.79	-	Yes
22	DETECTED	169.85	173.78	DETECTED	169.91	173.95	Yes
23	NOT DETECTED	169.80	-	NOT DETECTED	170.02	-	Yes
24	NOT DETECTED	169.73	-	NOT DETECTED	170.13	-	Yes
25	DETECTED	169.72	173.64	DETECTED	169.94	173.87	Yes
26	DETECTED	170.00	173.89	DETECTED	170.03	173.96	Yes
27	NOT DETECTED	169.87	-	NOT DETECTED	169.86	-	Yes
28	NOT DETECTED	169.86	-	NOT DETECTED	169.76	-	Yes
29	NOT DETECTED	169.89	-	NOT DETECTED	169.78	-	Yes
30	NOT DETECTED	169.98	-	NOT DETECTED	169.9	-	Yes
31**	NOT DETECTED	-	-	NOT DETECTED	-	-	Yes
32	DETECTED	170.06	174.05	DETECTED	169.89	173.92	Yes
33	NOT DETECTED	169.94	-	NOT DETECTED	169.93	-	Yes

Sample	*NPM1* singleplex	WT Allele Size	Mutant Allele Size	*NPM1* multiplex	WT Allele Size	Mutant Allele Size	Concordance
34	NOT DETECTED	169.86	-	NOT DETECTED	169.78	-	Yes
35	NOT DETECTED	169.90	-	NOT DETECTED	169.98	-	Yes
36	NOT DETECTED	169.84	-	NOT DETECTED	170.01	-	Yes
37	DETECTED	169.94	173.93	DETECTED	169.9	173.83	Yes
38	NOT DETECTED	169.98	-	NOT DETECTED	169.94	-	Yes
39	NOT DETECTED	169.97	-	NOT DETECTED	170.06	-	Yes
40	NOT DETECTED	170.05	-	NOT DETECTED	169.89	-	Yes

Abbreviations: WT, wild-type; * synthetic *NPM1* mutant fragment (no WT allele); ** synthetic *FLT3*/ITD mutant fragment (no WT allele).

2.2 Performance Evaluation: Precision

- **Test:** Intra and inter-assay coefficient of variation (%);
- **Result:** The coefficients of variation for intra-assay and inter-assay tests were 5.53% and 6.75%, respectively (Table S5).
- **Comments and interpretation:**

A 100% concordance rate was observed for the detection of *FLT3*/ITD and *NPM1* mutations in both intra-assay and inter-assay multiplex reactions (Table S6). Among the 40 samples tested, 22 samples showed *FLT3*/ITD mutations. Of these, one sample showed discordant allelic burden classification (high vs. low) in the inter-assay analysis. However, signal ratio values for this sample were close to the cutoff value (0.5), which may explain the classification difference, particularly considering the low standard deviation observed for intra-assay and inter-assay measurements (0.024 and 0.053, respectively) (Table S5). The coefficient of variation values indicate homogeneous data distribution, with only a few results exceeding 15% (classified as moderate dispersion), which was still considered acceptable: 15.34% (intra-assay test); 23.21% and 17.41% (inter-assay test).

Table S5. Results obtained in intra-assay and inter-assay analyses for concordance assessment of the multiplex PCR–capillary electrophoresis assay in the detection of *FLT3*/ITD and *NPM1* mutations for precision performance evaluation.

	**Mean standard deviation**	**Mean coefficient of variation (%)**	**Coefficient of Variation (%) Min-Max**
**Intra-assay**	0.024	5.534	0.474-15.342
**Inter-assay**	0.053	6.755	0.082-23.210

Table S6. Results obtained in intra-assay and inter-assay analyses for concordance assessment of the multiplex PCR–capillary electrophoresis assay in detection of *FLT3*/ITD and *NPM1* mutations for precision performance evaluation.

Sample	Date (dd/mm/yyyy)	*FLT3*/ITD									Intra-assay test			Inter-assay test			*NPM1*			Concord.
		Result	WT Allele Size	WT Allele Area	Mut. Allele 1—Size	Mut. Allele 1—Area	Mut. Allele 2—Size	Mut. Allele 2—Area	SR	AB	SR Mean	SD	CV (%)	SD	SR Mean	CV (%)	Result	WT Allele Size	Mut. Allele Size	
1	25/08/2023	NOT DETECTED	328.73	16836.2	-	-	-	-	-	-	-	-	-	-	-	-	DETECTED	169.81	173.74	
	12/09/2023	NOT DETECTED	328.64	6346.86	-	-	-	-	-	-	-	-	-	-	-	-	DETECTED	169.55	173.51	Yes
	12/09/2023	NOT DETECTED	328.65	5994.25	-	-	-	-	-	-	-	-	-	-	-	-	DETECTED	169.55	173.51	
2	25/08/2023	NOT DETECTED	328.69	12855.36	-	-	-	-	-	-	-	-	-	-	-	-	DETECTED	169.81	173.81	
	12/09/2023	NOT DETECTED	328.71	10799.16	-	-	-	-	-	-	-	-	-	-	-	-	DETECTED	169.51	173.57	Yes
	12/09/2023	NOT DETECTED	328.67	9610.55	-	-	-	-	-	-	-	-	-	-	-	-	DETECTED	169.65	173.6	
3	25/08/2023	DETECTED	328.73	6326.14	384.37	3111.25	-	-	0.49	LOW	-	-	-				DETECTED	169.77	173.66	
	12/09/2023	DETECTED	328.66	4074.63	384.62	2148.64	-	-	0.53	HIGH	0.53	0.003	0.617	0.023	0.51	4.621	DETECTED	169.52	173.52	Yes
	12/09/2023	DETECTED	328.66	4748.82	384.62	2482.4	-	-	0.52	HIGH							DETECTED	169.55	173.44	
4	25/08/2023	NOT DETECTED	328.69	8019.17	-	-	-	-	-	-	-	-	-	-	-	-	DETECTED	169.93	173.81	
	12/09/2023	NOT DETECTED	328.73	8515.88	-	-	-	-	-	-	-	-	-	-	-	-	DETECTED	169.63	173.69	Yes
	12/09/2023	NOT DETECTED	328.56	6891.48	-	-	-	-	-	-	-	-	-	-	-	-	DETECTED	169.55	173.51	
5	25/08/2023	DETECTED	328.85	3772.72	393.45	6874.47	-	-	1.82	HIGH	-	-	-				DETECTED	169.89	173.92	
	12/09/2023	DETECTED	328.71	2337.38	393.35	3934.47	-	-	1.68	HIGH	1.71	0.040	2.316	0.078	1.77	4.436	DETECTED	169.49	173.55	Yes
	12/09/2023	DETECTED	328.46	2575.68	393.34	4479.97	-	-	1.74	HIGH							DETECTED	169.63	173.62	
6	25/08/2023	DETECTED	328.78	8547.82	353.03	1221.99	-	-	0.14	LOW	-	-	-				DETECTED	169.98	174.01	
	12/09/2023	DETECTED	328.55	7188.16	352.82	1003.25	-	-	0.14	LOW	0.14	0.003	1.874	0.004	0.14	2.627	DETECTED	169.55	173.63	Yes
	12/09/2023	DETECTED	328.50	2738.52	352.84	372.22	-	-	0.14	LOW							DETECTED	169.47	173.57	
7	25/08/2023	DETECTED	328.76	20347.38	352.82	1438.57	-	-	0.07	LOW	-	-	-				DETECTED	170.01	173.92	
	12/09/2023	DETECTED	328.65	7969.54	352.72	459.77	-	-	0.06	LOW	0.06	0.001	1.394	0.009	0.06	13.636	DETECTED	169.55	173.51	Yes
	12/09/2023	DETECTED	328.57	14238.64	352.74	837.8	-	-	0.06	LOW							DETECTED	169.49	173.48	
8	25/08/2023	NOT DETECTED	328.68	14969.34	-	-	-	-	-	-	-	-	-	-	-	-	DETECTED	170.06	173.94	
	12/09/2023	NOT DETECTED	328.57	8896.77	-	-	-	-	-	-	-	-	-	-	-	-	DETECTED	169.63	173.57	Yes
	12/09/2023	NOT DETECTED	328.6400	13717.63	-	-	-	-	-	-	-	-	-	-	-	-	DETECTED	169.55	173.51	
9	25/08/2023	NOT DETECTED	328.62	11863.3	-	-	-	-	-	-	-	-	-	-	-	-	DETECTED	169.83	173.78	
	12/09/2023	NOT DETECTED	328.6	13002.17	-	-	-	-	-	-	-	-	-	-	-	-	DETECTED	169.47	173.45	Yes
	12/09/2023	NOT DETECTED	328.59	11213.76	-	-	-	-	-	-	-	-	-	-	-	-	DETECTED	169.47	173.45	
10	25/08/2023	NOT DETECTED	328.68	12774.06	-	-	-	-	-	-	-	-	-	-	-	-	DETECTED	169.85	173.88	
	12/09/2023	NOT DETECTED	328.62	11990.02	-	-	-	-	-	-	-	-	-	-	-	-	DETECTED	169.61	173.59	Yes
	12/09/2023	NOT DETECTED	328.51	11331.64	-	-	-	-	-	-	-	-	-	-	-	-	DETECTED	169.53	173.6	
11	25/08/2023	DETECTED	328.7	11640.75	352.88	1056.11	420.66	505.8	0.13	LOW	-	-	-	0.021	0.12	17.412	DETECTED	169.79	173.76	Yes
	12/09/2023	DETECTED	328.66	8635.54	352.85	553.52	420.74	303.3	0.10	LOW	0.10	0.008	7.483				DETECTED	169.53	173.52	

Sample	Date (dd/mm/yyyy)	*FLT3*/ITD										Intra-assay test			Inter-assay test			*NPM1*			Concord.
		Result	WT Allele Size	WT Allele Area	Mut. Allele 1—Size	Mut. Allele 1—Area	Mut. Allele 2—Size	Mut. Allele 2—Area	SR	AB	SR Mean	SD	CV (%)	SD	SR Mean	CV (%)	Result	WT Allele Size	Mut. Allele Size	
	12/09/2023	DETECTED	328.65	9746.44	352.97	689.48	420.62	385.6	0.11	LOW							DETECTED	169.49	173.48	
	25/08/2023	NOT DETECTED	-	-	-	-	-	-	-	-	-	-	-	-	-	-	DETECTED	-	174.12	
12*	12/09/2023	NOT DETECTED	-	-	-	-	-	-	-	-	-	-	-	-	-	-	DETECTED	-	173.79	Yes
	12/09/2023	NOT DETECTED	-	-	-	-	-	-	-	-	-	-	-	-	-	-	DETECTED	-	173.35	
	25/08/2023	DETECTED	328.53	8240.18	355.94	4152.96	-	-	0.50	HIGH	-	-	-				DETECTED	169.88	173.8	
13	12/09/2023	DETECTED	328.65	3604.76	355.68	1736.25	-	-	0.48	LOW	0.50	0.032	6.424	0.000	0.50	0.082	DETECTED	169.59	173.55	Yes
	12/09/2023	DETECTED	328.56	5082.66	355.67	2681.06	-	-	0.53	HIGH							DETECTED	169.65	173.6	
	25/08/2023	DETECTED	328.59	6830.35	396.31	2144.40	-	-	0.31	LOW	-	-	-				NOT DETECTED	169.92	-	
14	12/09/2023	DETECTED	328.59	3355.07	396.17	805.36	-	-	0.24	LOW	0.26	0.034	12.876	0.035	0.29	12.200	NOT DETECTED	169.51	-	Yes
	12/09/2023	DETECTED	328.65	5522.65	396.16	1591.25	-	-	0.29	LOW							NOT DETECTED	169.59	-	
	25/08/2023	DETECTED	328.62	6427.64	370.41	866.65	396.20	798.7	0.26	LOW	-	-	-				NOT DETECTED	169.91	-	
15	12/09/2023	DETECTED	328.6	4330.01	370.11	456.15	396.01	375.5	0.19	LOW	0.21	0.030	13.927	0.033	0.24	13.795	NOT DETECTED	169.63	-	Yes
	12/09/2023	DETECTED	328.5	5949.12	370.25	762.9	396.15	629.3	0.23	LOW							NOT DETECTED	169.58	-	
	25/08/2023	DETECTED	328.58	10768.61	387.57	4346.71	-	-	0.40	LOW	-	-	-				NOT DETECTED	169.74	-	
16	12/09/2023	DETECTED	328.65	3134.79	387.48	990.64	-	-	0.32	LOW	0.35	0.051	14.436	0.037	0.38	9.678	NOT DETECTED	169.55	-	Yes
	12/09/2023	DETECTED	328.55	8152.9	387.4	3162.21	-	-	0.39	LOW							NOT DETECTED	169.65	-	
	25/08/2023	DETECTED	328.74	7297.69	375.99	4634.08	-	-	0.64	HIGH	-	-	-				NOT DETECTED	169.81	-	
17	12/09/2023	DETECTED	328.61	5133.5	375.9	3018.33	-	-	0.59	HIGH	0.59	0.007	1.198	0.030	0.61	4.839	NOT DETECTED	169.55	-	Yes
	12/09/2023	DETECTED	328.59	4169.57	375.83	2493.47	-	-	0.60	HIGH							NOT DETECTED	169.57	-	
	25/08/2023	DETECTED	328.53	10301.50	358.61	4504.58	-	-	0.44	LOW	-	-	-				NOT DETECTED	170.00	-	
18	12/09/2023	DETECTED	328.67	6774.37	358.5	3027.71	-	-	0.45	LOW	0.44	0.013	2.915	0.000	0.44	0.103	NOT DETECTED	169.51	-	Yes
	12/09/2023	DETECTED	328.53	7451.74	358.4	3195.93	-	-	0.43	LOW							NOT DETECTED	169.56	-	
	25/08/2023	DETECTED	328.57	13937.23	382.03	780.19	-	-	0.06	LOW	-	-	-				NOT DETECTED	169.88	-	
19	12/09/2023	DETECTED	328.59	7143.37	381.77	375.85	-	-	0.05	LOW	0.05	0.003	5.596	0.001	0.06	1.527	NOT DETECTED	169.61	-	Yes
	12/09/2023	DETECTED	328.53	8665.38	381.83	493.5	-	-	0.06	LOW							NOT DETECTED	169.49	-	
	25/08/2023	DETECTED	328.56	7912.83	364.64	6144.33	-	-	0.78	HIGH	-	-	-				DETECTED	169.88	173.8	
20	12/09/2023	DETECTED	328.68	3891.66	364.5	3003.05	-	-	0.77	HIGH	0.79	0.020	2.536	0.007	0.78	0.838	DETECTED	169.65	173.6	Yes
	12/09/2023	DETECTED	328.56	6655.88	364.38	5323.69	-	-	0.80	HIGH							DETECTED	169.52	173.47	
	25/08/2023	DETECTED	328.5	6108.19	347.02	4103.69	-	-	0.67	HIGH	-	-	-				NOT DETECTED	169.79	-	
21	12/09/2023	DETECTED	328.49	4600.06	347.07	3271.12	-	-	0.71	HIGH	0.70	0.016	2.299	0.020	0.69	2.876	NOT DETECTED	169.55	-	Yes
	12/09/2023	DETECTED	328.66	5656.17	346.99	3893.46	-	-	0.69	HIGH							NOT DETECTED	169.55	-	
	25/08/2023	DETECTED	328.53	7765.24	358.59	3804.15	-	-	0.49	LOW	-	-	-				DETECTED	169.91	173.95	
22	12/09/2023	DETECTED	328.49	6502.29	358.53	3091.03	-	-	0.48	LOW	0.48	0.002	0.474	0.009	0.48	1.890	DETECTED	169.58	173.54	Yes
	12/09/2023	DETECTED	328.5	5530.02	358.53	2646.52	-	-	0.48	LOW							DETECTED	169.61	173.58	
23	25/08/2023	DETECTED	328.62	1613.68	393.14	10431.25	-	-	6.46	HIGH	-	-	-	0.573	6.06	9.458	NOT DETECTED	170.02	-	Yes

Sample	Date (dd/mm/yyyy)	*FLT3*/ITD									Intra-assay test			Inter-assay test			*NPM1*			Concord.
		Result	WT Allele Size	WT Allele Area	Mut. Allele 1—Size	Mut. Allele 1—Area	Mut. Allele 2—Size	Mut. Allele 2—Area	SR	AB	SR Mean	SD	CV (%)	SD	SR Mean	CV (%)	Result	WT Allele Size	Mut. Allele Size	
	12/09/2023	DETECTED	328.5	999.49	393.11	5702.65	-	-	5.71	HIGH	5.65	0.073	1.293				NOT DETECTED	169.57	-	
	12/09/2023	DETECTED	328.5	1361.25	393.13	7625.93	-	-	5.60	HIGH							NOT DETECTED	169.45	-	
24	25/08/2023	DETECTED	328.59	9152.08	396.41	4118.18	-	-	0.45	LOW	-	-	-	0.011	0.46	2.318	NOT DETECTED	170.13	-	Yes
	12/09/2023	DETECTED	328.56	5699.03	396.28	2544.36	-	-	0.45	LOW	0.46	0.026	5.632				NOT DETECTED	169.53	-	
	12/09/2023	DETECTED	328.59	4208.41	396.28	2034.71	-	-	0.48	LOW							NOT DETECTED	169.56	-	
25	25/08/2023	DETECTED	328.5	11388.98	358.5	682.59	-	-	0.06	LOW	-	-	-	0.000	0.06	0.707	DETECTED	169.94	173.87	Yes
	12/09/2023	DETECTED	328.49	11435.42	358.43	706.4	-	-	0.06	LOW	0.06	0.002	2.888				DETECTED	169.58	173.54	
	12/09/2023	DETECTED	328.65	8482.04	358.4	502.99	-	-	0.06	LOW							DETECTED	169.58	173.54	
26	25/08/2023	DETECTED	328.53	15447.35	358.73	1047.5	454.11	837.2	0.12	LOW	-	-	-	0.002	0.12	1.876	DETECTED	170.03	173.96	Yes
	12/09/2023	DETECTED	328.55	12362.53	358.61	867.8	454.04	655.6	0.12	LOW	0.12	0.006	5.257				DETECTED	169.53	173.6	
	12/09/2023	DETECTED	328.55	10447.36	358.61	818.51	454.07	376.6	0.11	LOW							DETECTED	169.63	173.56	
27	25/08/2023	DETECTED	328.57	15302.56	454.14	2044.99	-	-	0.13	LOW	-	-	-	0.027	0.11	23.210	NOT DETECTED	169.86	-	Yes
	12/09/2023	DETECTED	328.56	9821.95	454.07	1044.72	-	-	0.11	LOW	0.10	0.015	15.342				NOT DETECTED	169.47	-	
	12/09/2023	DETECTED	328.59	9260.54	454.05	792.21	-	-	0.09	LOW							NOT DETECTED	169.47	-	
28	25/08/2023	DETECTED	328.44	10115.97	358.7	1803.41	-	-	0.18	LOW	-	-	-	0.021	0.16	12.933	NOT DETECTED	169.76	-	Yes
	12/09/2023	DETECTED	328.49	6439.48	358.6	1025.01	-	-	0.16	LOW	0.15	0.015	10.270				NOT DETECTED	169.63	-	
	12/09/2023	DETECTED	328.55	4877.77	358.5	671.29	-	-	0.14	LOW							NOT DETECTED	169.49	-	
29	25/08/2023	NOT DETECTED	328.53	11192.76	-	-	-	-	-	-	-	-	-	-	-	-	NOT DETECTED	169.78	-	Yes
	12/09/2023	NOT DETECTED	328.56	7396.65	-	-	-	-	-	-	-	-	-	-	-	-	NOT DETECTED	169.65	-	
	12/09/2023	NOT DETECTED	328.65	6893.82	-	-	-	-	-	-	-	-	-	-	-	-	NOT DETECTED	169.56	-	
30	25/08/2023	DETECTED	328.53	2061.05	358.62	6498.15	-	-	3.15	HIGH	-	-	-	0.226	2.99	7.558	NOT DETECTED	169.9	-	Yes
	12/09/2023	DETECTED	328.65	1439.15	358.59	3941.65	-	-	2.74	HIGH	2.83	0.133	4.695				NOT DETECTED	169.55	-	
	12/09/2023	DETECTED	328.53	1196.58	358.51	3502.38	-	-	2.93	HIGH							NOT DETECTED	169.55	-	
31**	25/08/2023	DETECTED	-	-	351.55	33015.99	-	-	-	-	-	-	-	-	-	-	NOT DETECTED	-	-	Yes
	12/09/2023	DETECTED	-	-	351.25	25069.3	-	-	-	-	-	-	-	-	-	-	NOT DETECTED	-	-	
	12/09/2023	DETECTED	-	-	351.25	28681.54	-	-	-	-	-	-	-	-	-	-	NOT DETECTED	-	-	
32	25/08/2023	NOT DETECTED	328.56	16944.26	-	-	-	-	-	-	-	-	-	-	-	-	DETECTED	169.89	173.92	Yes
	12/09/2023	NOT DETECTED	328.58	6344.4	-	-	-	-	-	-	-	-	-	-	-	-	DETECTED	169.5	173.56	
	12/09/2023	NOT DETECTED	328.58	10150.4	-	-	-	-	-	-	-	-	-	-	-	-	DETECTED	169.5	173.56	
33	25/08/2023	NOT DETECTED	328.47	18660.43	-	-	-	-	-	-	-	-	-	-	-	-	NOT DETECTED	169.93	-	Yes
	12/09/2023	NOT DETECTED	328.62	15985.79	-	-	-	-	-	-	-	-	-	-	-	-	NOT DETECTED	169.55	-	
	12/09/2023	NOT DETECTED	328.49	15495.13	-	-	-	-	-	-	-	-	-	-	-	-	NOT DETECTED	169.55	-	
34	25/08/2023	NOT DETECTED	328.53	14179.29	-	-	-	-	-	-	-	-	-	-	-	-	NOT DETECTED	169.78	-	Yes
	12/09/2023	NOT DETECTED	328.65	11773.1	-	-	-	-	-	-	-	-	-	-	-	-	NOT DETECTED	169.66	-	
	12/09/2023	NOT DETECTED	328.54	10283.99	-	-	-	-	-	-	-	-	-	-	-	-	NOT DETECTED	169.63	-	

Sample	Date (dd/mm/yyyy)	*FLT3*/ITD									Intra-assay test			Inter-assay test			*NPM1*			Concord.
		Result	WT Allele Size	WT Allele Area	Mut. Allele 1—Size	Mut. Allele 1—Area	Mut. Allele 2—Size	Mut. Allele 2—Area	SR	AB	SR Mean	SD	CV (%)	SD	SR Mean	CV (%)	Result	WT Allele Size	Mut. Allele Size	
35	25/08/2023	NOT DETECTED	328.45	8062.27	-	-	-	-	-	-	-	-	-	-	-	-	NOT DETECTED	169.98	-	Yes
	12/09/2023	NOT DETECTED	328.56	3433.98	-	-	-	-	-	-	-	-	-	-	-	-	NOT DETECTED	169.63	-	
	12/09/2023	NOT DETECTED	328.59	2929.27	-	-	-	-	-	-	-	-	-	-	-	-	NOT DETECTED	169.47	-	
36	25/08/2023	NOT DETECTED	328.56	10295.86	-	-	-	-	-	-	-	-	-	-	-	-	NOT DETECTED	170.01	-	Yes
	12/09/2023	NOT DETECTED	328.58	9237.09	-	-	-	-	-	-	-	-	-	-	-	-	NOT DETECTED	169.66	-	
	12/09/2023	NOT DETECTED	328.61	3571.73	-	-	-	-	-	-	-	-	-	-	-	-	NOT DETECTED	169.63	-	
37	25/08/2023	NOT DETECTED	328.54	9441.42	-	-	-	-	-	-	-	-	-	-	-	-	DETECTED	169.9	173.83	Yes
	12/09/2023	NOT DETECTED	328.52	7509.96	-	-	-	-	-	-	-	-	-	-	-	-	DETECTED	169.56	173.63	
	12/09/2023	NOT DETECTED	328.68	732.61	-	-	-	-	-	-	-	-	-	-	-	-	DETECTED	169.66	173.6	
38	25/08/2023	NOT DETECTED	328.53	9254.16	-	-	-	-	-	-	-	-	-	-	-	-	NOT DETECTED	169.94	-	Yes
	12/09/2023	NOT DETECTED	328.52	6971.98	-	-	-	-	-	-	-	-	-	-	-	-	NOT DETECTED	169.45	-	
	12/09/2023	NOT DETECTED	328.68	4660.92	-	-	-	-	-	-	-	-	-	-	-	-	NOT DETECTED	169.56	-	
39	25/08/2023	NOT DETECTED	328.51	6131.19	-	-	-	-	-	-	-	-	-	-	-	-	NOT DETECTED	170.06	-	Yes
	29/09/2023	NOT DETECTED	328.92	9071.06	-	-	-	-	-	-	-	-	-	-	-	-	NOT DETECTED	169.77	-	
	29/09/2023	NOT DETECTED	328.83	7278.71	-	-	-	-	-	-	-	-	-	-	-	-	NOT DETECTED	169.78	-	
40	25/08/2023	NOT DETECTED	328.53	12361.92	-	-	-	-	-	-	-	-	-	-	-	-	NOT DETECTED	169.89	-	Yes
	12/09/2023	NOT DETECTED	328.58	11187.38	-	-	-	-	-	-	-	-	-	-	-	-	NOT DETECTED	169.66	-	
	12/09/2023	NOT DETECTED	328.57	5121.61	-	-	-	-	-	-	-	-	-	-	-	-	NOT DETECTED	169.59	-	

Abbreviations: WT, wild-type; mut., mutant; SR, signal ratio; AB, allelic burden; SD, standard deviation; CV, coefficient of variation; Concord., concordance; * synthetic *NPM1* mutant fragment (no WT allele); ** synthetic *FLT3*/ITD mutant fragment (no WT allele).

2.3 Performance Evaluation: In silico Specificity

- **Test:** Percent identity;
- **Result:** 100% identity;
- **Comments and interpretation:**

The primers used in the assay ranged from 23 to 32 base pairs in length, with melting temperatures (Tm) between 60.0 and 65.05 °C (maximum variation of 5 °C between primer pairs) and GC content ranging from 25.00% to 53.85% (Table S7). The expected amplified fragment sizes for mutation detection in the *NPM1* and *FLT3*/ITD genes are approximately 172 bp (Figures 1 and 3A) and 329 bp (Figures 2 and 3B), respectively.

For the *NPM1* gene, in silico analysis showed no off-target amplification (Figure 1). Primer annealing occurs in exon 11 of the gene (current reference transcript NM_002520; corresponding to exon 12 in the previous reference transcript NM_001355006), which represents the region with the highest prevalence of *NPM1* mutations (Table S8). The amplified fragment generated for *NPM1* mutation detection (Figure 3A) was aligned with the wild-type reference sequence, resulting in 100% identity, indicating high primer specificity. Alignment with mutated sequences (types A, B and D) showed 98% identity, with the remaining 2% corresponding to the nucleotide insertion associated with the mutation (Figure 4).

For the *FLT3*/ITD gene, in silico analysis indicated that the primers could potentially anneal to sequences other than the reference sequence (RefSeq NG_007066.1); however, these correspond to alternative splice variants of the same gene (Figure 2). Because the mutations investigated in the *FLT3* gene are internal tandem duplications (ITD), the number of inserted nucleotides may vary considerably within exons 14 and 15. Therefore, the primer annealing regions were specifically evaluated in silico to confirm coverage of these exons, which was verified using the NCBI BLAST software (Figure 2). In addition, alignment of the amplified fragment generated by the assay with a wild-type *FLT3* sequence confirmed 100% identity (Figure 5).

Table S7. General information on the primers used in the multiplex assay for detection of *FLT3*/ITD and *NPM1* mutations.

	Sequence (5′ -> 3′)	Size (bp)	Tm (°C)	%GC
***NPM1* Forward**	ATTTCTTTTTTTTTTTTTCCAGGCTATTCAAG	32	60.00	25.00
***NPM1* Reverse**	HEX-CACGGTAGGGAAAGTTCTCACTCTGC	26	65.05	53.85
***FLT3*/ITD Forward**	GCAATTTAGGTATGAAAGCCAGC	23	58.64	43.48
***FLT3*/ITD Reverse**	FAM-CTTTCAGCATTTTGACGGCAACC	23	61.64	47.83

Abbreviations: bp, base pairs; Tm, melting temperature.

Table S8. Most prevalent *NPM1* gene mutations, corresponding nucleotide insertions and GenBank accession numbers of the reference sequences (RefSeq).

Mutation Type	Nucleotide insertion position	RefSeq
No mutation (wild-type)	-	NG_016018.1
Type A mutation	c. 860_863dupTCTG	AY740634
Type B mutation	c. 862_863insCATG	AY740635
Type D mutation	c. 863_864insCCTG	AY740637

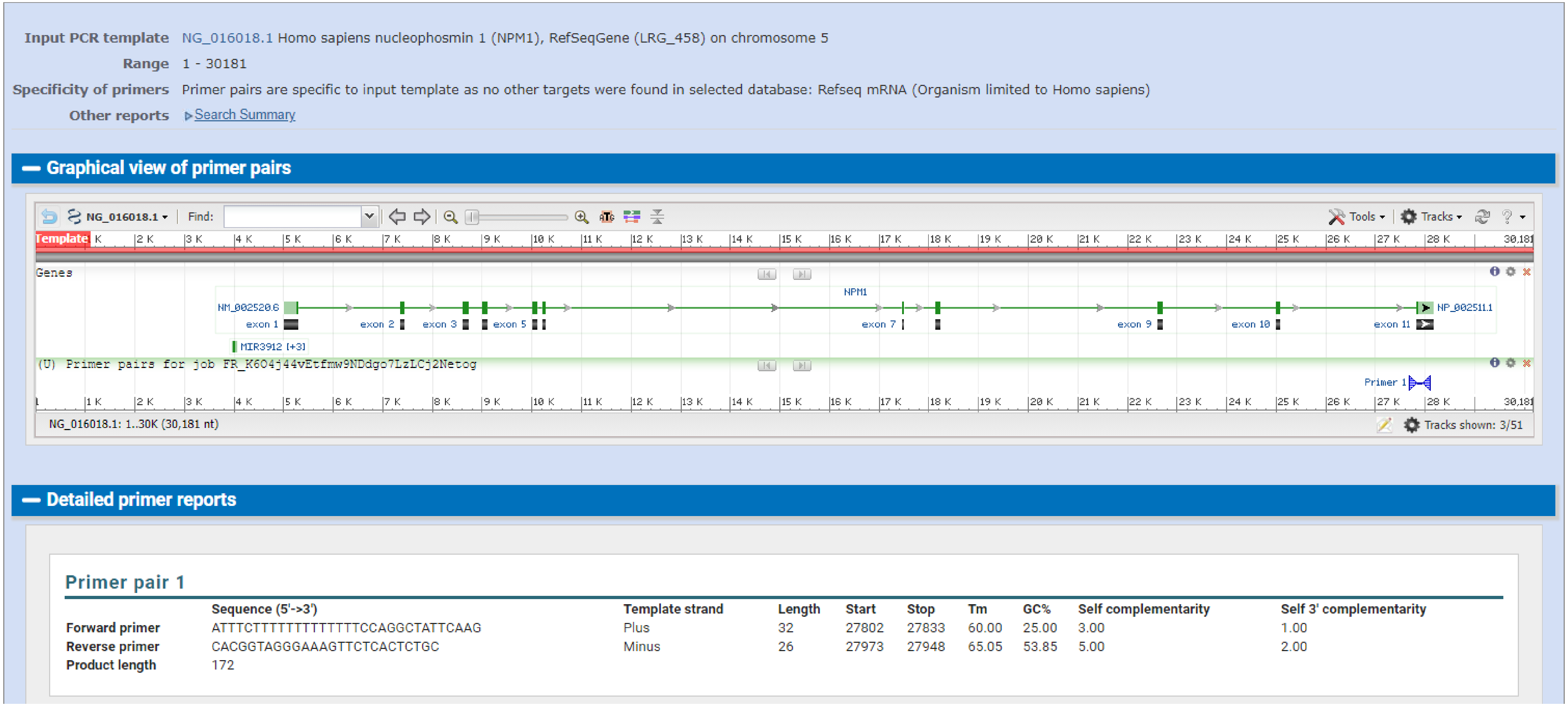

Figure S1. In silico primer specificity analysis for *NPM1* mutation detection (RefSeq NG_016018.1), targeting exon 11, performed using NCBI Primer-BLAST.

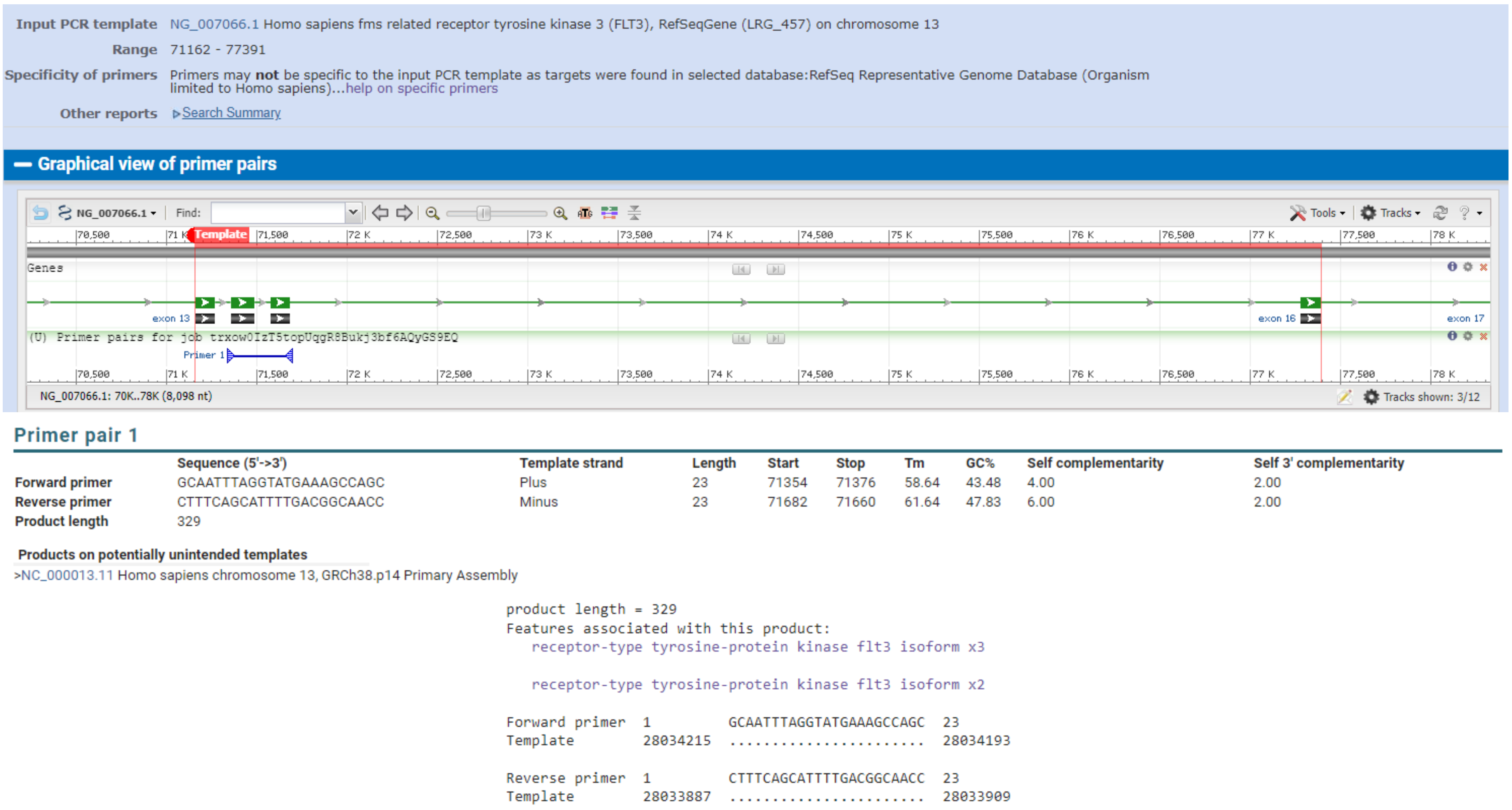

Figure S2. In silico primer specificity analysis for *FLT3*/ITD mutation detection (RefSeq NG_007066.1), targeting exons 14 and 15, performed using NCBI Primer-BLAST.

FASTA ▾ Send to: ▾

(A)

Homo sapiens nucleophosmin 1 (NPM1), RefSeqGene (LRG_458) on chromosome 5

NCBI Reference Sequence: NG_016018.1

GenBank Graphics

```
>NG_016018.1:27802-27980 Homo sapiens nucleophosmin 1 (NPM1), RefSeqGene (LRG_458) on
chromosome 5
ATTTCTTTTTTTTTTTTTCCAGGCTATTCAAGATCTCTGGCAGTGGAGGAAGTCTCTTTAAGAAAATAGT
TTAAACAATTTGTTAAAAAATTTTCCGTCTTATTTCATTTCTGTAACAGTTGATATCTGGCTGTCCTTTT
TATAATGCAGAGTGAGAACTTTCCCTACCGTGTTTGATA
```

Homo sapiens fms related receptor tyrosine kinase 3 (FLT3), RefSeqGene (LRG_457) on chromosome 13

NCBI Reference Sequence: NG_007066.1

GenBank Graphics

```
>NG_007066.1:71354-71682 Homo sapiens fms related receptor tyrosine kinase 3 (FLT3),
RefSeqGene (LRG_457) on chromosome 13
GCAATTTAGGTATGAAAGCCAGCTACAGATGGTACAGGTGACCGGCTCCTCAGATAATGAGTACTTCTAC
GTTGATTTCAGAGAATATGAATATGATCTCAAATGGGAGTTTCCAAGAGAAAATTTAGAGTTTGGTAAGA
ATGGAATGTGCCAAATGTTTCTGCAGCATTTCTTTTCCATTGGAAAATCTTTAAAATGCACGTACTCACC
ATTTGTCTTTGCAGGGAAGGTACTAGGATCAGGTGCTTTTGGAAAAGTGATGAACGCAACAGCTTATGGA
ATTAGCAAAACAGGAGTCTCAATCCAGGTTGCCGTCAAAATGCTGAAAG
```

(B)

Figure S3. (A) Nucleotide sequence of the amplified fragment obtained from alignment of the primer pair targeting the *NPM1* gene. **(B)** Nucleotide sequence of the amplified fragment obtained from alignment of the primer pair targeting the *FLT3* gene. Generated using NCBI GenBank.

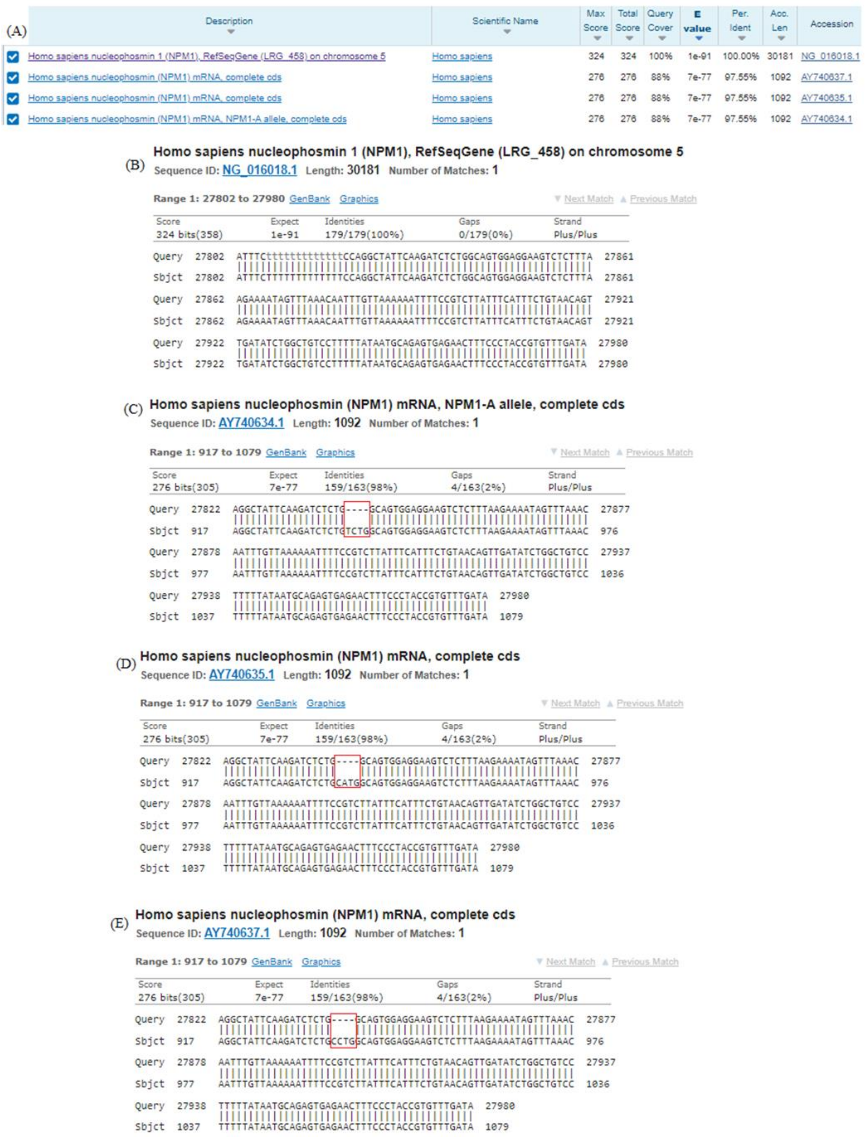

Figure S4. **(A)** General alignment information comparing the amplified fragment (query) with different *NPM1* mutation sequences (subject). **(B)** Alignment of the amplified fragment with the wild-type *NPM1* sequence, showing 100% identity. **(C)** Alignment of the amplified fragment with a type A *NPM1* mutation sequence, showing 98% identity, with the remaining 2% corresponding to the insertion gap of four base pairs (TCTG). **(D)** Alignment of the amplified fragment with a type B *NPM1* mutation sequence, showing 98% identity, with the remaining 2% corresponding to the insertion gap of four base pairs (CATG). **(E)** Alignment of the amplified fragment with a type D *NPM1* mutation sequence, showing 98% identity, with the remaining 2% corresponding to the insertion gap of four base pairs (CCTG).

(A)

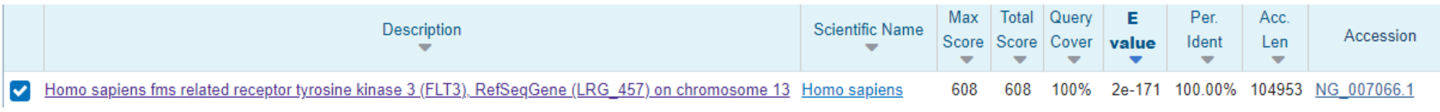

Description	Scientific Name	Max Score	Total Score	Query Cover	E value	Per. Ident	Acc. Len	Accession
Homo sapiens fms related receptor tyrosine kinase 3 (FLT3), RefSeqGene (LRG_457) on chromosome 13	Homo sapiens	608	608	100%	2e-171	100.00%	104953	NG_007066.1

(B)

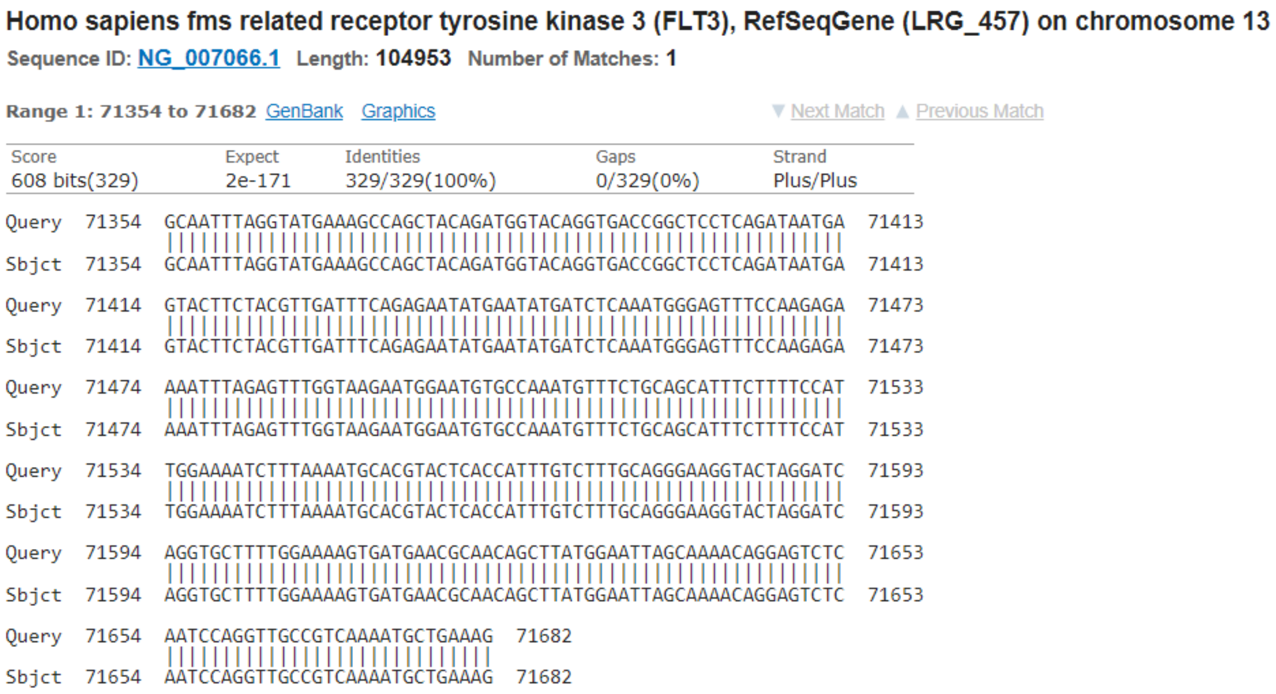

Figure S5. (A) General alignment information of the amplified fragment (query) with the sequence showing the highest identity percentage. **(B)** Alignment of the amplified fragment generated using the primer pair targeting exons 14 and 15 of the *FLT3* gene with the wild-type sequence, showing 100% identity.

3. Conclusion

Based on the results obtained in the analytical validation tests, the multiplex PCR followed by fragment analysis for detection of *FLT3*/ITD and *NPM1* mutations demonstrated adequate analytical performance in terms of specificity, concordance and precision under the conditions evaluated. The validation results support the implementation of the assay for routine molecular diagnostic use in the laboratory, within the scope of AML mutation screening and risk stratification. As this is an in-house assay, laboratories adopting this protocol should perform local validation according to their institutional quality management and accreditation requirements.
